# Dissecting the complex genetic basis of pre- and post-harvest traits in *Vitis vinifera L*. using genome-wide association studies

**DOI:** 10.1093/hr/uhad283

**Published:** 2024-01-03

**Authors:** Julian García-Abadillo, Paola Barba, Tiago Carvalho, Viviana Sosa-Zuñiga, Roberto Lozano, Humberto Fanelli Carvalho, Miguel Garcia-Rojas, Erika Salazar, Julio Isidro y Sánchez

**Affiliations:** Centro de Biotecnología y Genómica de Plantas, Universidad Politécnica de Madrid (UPM) - Instituto Nacional de Investigación y Tecnología Agraria y Alimentaria (INIA), Campus de Montegancedo - Pozuelo de Alarcón, 28223, Madrid, Spain; Genetic Resources Unit and Germplasm Bank, La Platina, Instituto de Investigaciones Agropecuarias, Av Santa Rosa 11610, La pintana, Santiago, Chile; Sun World International, 28994 Gromer Av, Wasco, 93280, California, USA; Freelance, Madrid, Spain; Instituto de Ciencias Químicas y Aplicadas (ICQA), Universidad Autónoma de Chile, El Llano Subercaseaux 2801, Santiago, Chile; Ginkgo Bioworks, Boston, Massachusetts, USA; Centro de Biotecnología y Genómica de Plantas, Universidad Politécnica de Madrid (UPM) - Instituto Nacional de Investigación y Tecnología Agraria y Alimentaria (INIA), Campus de Montegancedo - Pozuelo de Alarcón, 28223, Madrid, Spain; Genetic Resources Unit and Germplasm Bank, La Platina, Instituto de Investigaciones Agropecuarias, Av Santa Rosa 11610, La pintana, Santiago, Chile; Genetic Resources Unit and Germplasm Bank, La Platina, Instituto de Investigaciones Agropecuarias, Av Santa Rosa 11610, La pintana, Santiago, Chile; Centro de Biotecnología y Genómica de Plantas, Universidad Politécnica de Madrid (UPM) - Instituto Nacional de Investigación y Tecnología Agraria y Alimentaria (INIA), Campus de Montegancedo - Pozuelo de Alarcón, 28223, Madrid, Spain

## Abstract

Addressing the pressing challenges in agriculture necessitates swift advancements in breeding programs, particularly for perennial crops like grapevines. Moving beyond the traditional biparental quantitative trait loci (QTL) mapping, we conducted a genome-wide association study (GWAS) encompassing 588 *Vitis vinifera L.* cultivars from a Chilean breeding program, spanning three seasons and testing 13 key yield-related traits. A strong candidate gene, Vitvi11g000454, located on chromosome 11 and related to plant response to biotic and abiotic stresses through jasmonic acid signaling, was associated with berry width and holds potential for enhancing berry size in grape breeding. We also mapped novel QTL associated with post-harvest traits across chromosomes 2, 4, 9, 11, 15, 18, and 19, broadening our grasp on the genetic intricacies dictating fruit post-harvest behavior, including decay, shriveling, and weight loss. Leveraging gene ontology annotations, we drew parallels between traits and scrutinized candidate genes, laying a robust groundwork for future trait-feature identification endeavors in plant breeding. We also highlighted the importance of carefully considering the choice of the response variable in GWAS analyses, as the use of best linear unbiased estimators (BLUEs) corrections in our study may have led to the suppression of some common QTL in grapevine traits. Our results underscore the imperative of pioneering non-destructive evaluation techniques for long-term conservation traits, offering grape breeders and cultivators insights to improve post-harvest table grape quality and minimize waste.

## Introduction

Grapevine plays a pivotal role in global fruit production, generating almost 70 million tons of fruit annually, of which 43.3% is table grapes [1, 2]. The ideal grape qualities for consumers and growers encompass traits such as size, taste, firmness, and post-harvest longevity, while the economic value is intrinsically tied to yield and quality [3–7]. However, various factors including rachis browning, cluster decay, and berry cracking can compromise post-harvest quality, affecting not only the aesthetics but also the sensory perception of the fruits [8–13].

Traditional grapevine breeding methods face challenges in balancing these desirable traits, largely due to the considerable investment in time and resources, in addition to the prolonged juvenile phase characteristic of woody perennial species [14–17]. This issue is particularly pertinent since the fruit characteristics evaluation can only start after the plant matures, typically in the fourth or fifth year of its life cycle [18]. An emergent solution lies in employing molecular markers derived from quantitative trait loci (QTL) analysis. This approach facilitates the prediction of fruit characteristics in immature plants, and promises a substantial reduction in the breeding cycle of up to a decade, as well as notable cost savings of 16%–34% [19–21].

Due to the commercial importance of seedlessness in table grapes, further research has been carried out to elucidate the genetic architecture of this trait. Bouquet *et al*. [22] proposed the prevailing hypothesis on seedlessness’ genetic control, suggesting it is largely controlled by a dominant regulator gene, the seed development inhibitor (SDI), and three unidentified recessive genes. Chromosome 18 hosts a QTL linked to stenospermocarpic seedlessness, accounting for 50%–90% of seed weight variation [23, 24]. Further studies have identified VvAGL11, a major functional candidate gene encoding a MADS-box transcription factor involved in seed development [25]. Marker-assisted selection (MAS) suitable for use in grape breeding programs has been developed [26]. While additional QTL linked to seed dry weight have been found on chromosomes 2 [27], 5 [4, 27], and 14 [4, 27], their contributions are smaller compared to SDI. In terms of fresh weight, loci have been identified across numerous chromosomes [4, 24, 28–30]. Likewise, loci influencing seed number have been detected across various chromosomes [4, 24, 28–31].

Berry and seed traits in table grapes have shown a strong positive correlation [18]. Berry size, in particular, has been linked to the SDI locus with a major QTL, while additional minor QTL provides additive contributions [4, 32–34]. Berry weight loci reside in a range of chromosomes, including 1, 2, 4, 5, 7, 9, 10, 11, 13, 15, 16, and 18 [24, 28, 29, 31, 33, 35–39], while cluster weight is controlled by loci on chromosomes 2, 5, 10, 12, 16, 17, and 18 [36, 40].

The genetic basis of berry shape, defined as the ratio between width and height or based on categorical scales, has also been investigated [41, 42] and has been associated with genes on multiple chromosomes and a variety of functions, including transcription regulation, binding activities, catalytic activity, cell wall biogenesis, and protein transport [42]. Berry diameter is influenced by loci on chromosomes 2 and 18 [24, 33], whereas berry volume is impacted by loci on chromosomes 2, 12, 17, and 18 [33, 40]. Berry cracking has been linked to regions in chromosomes 11 and 13 [11, 35].

Linkage mapping has contributed significantly to our understanding of grapevine traits but has limitations due to a reliance on high frequency of recombination events [43]. Genome-wide association analysis (GWAS) provides an alternative by studying genetic architecture and gene interactions influencing traits [44–49]. Despite these advances, further GWAS studies are needed for berry diameter, cluster weight, and post-harvest traits, the genetic underpinnings of which remain less explored than those of seed traits.

Exploring trait genetic architecture requires diverse individuals representing existing trait variability [50]. Yet, many studies have relied on single-population analysis, underscoring the need for broader analyses, such as the three-population approach as demonstrated by [4].

In this study, we carried out a GWAS analysis on a comprehensive set of 588 genotypes, which includes seven populations and commercial varieties, to investigate into 13 yield-associated traits. Our main goals were i) to discover new QTL, with a special emphasis on the lesser studied post-harvest traits, and ii) to corroborate QTL previously pinpointed in other studies. Consequently, we aimed to elucidate the genetic architecture underpinning these traits.

Moreover, we present two novel statistical methods for the *in silico* validation of candidate single nucleotide polymorphisms (SNPs) that surpass the set *P*-value threshold. Firstly, we evaluated the suitability of molecular markers for MAS by integrating the ‘bagging’ concept [51]. In the second approach, we utilized Gene Ontology-derived bioinformatic data to construct a trait network based on correlations, efficiently elucidating genetic interrelationships among traits. This enables the discernment of underlying genetic relationships among traits in an efficient and parsimonious manner.

## Results

### Phenotype analysis

Figure 2A presents the adjusted phenotypes, which were tested for significant differences among families using the ANOVA test ($P<0.01$, Supplementary Table S3). While the distribution of all traits, except for the seed traits, approximated a normal distribution, the seed traits showed mixture of two Gaussian distributions. Despite our attempts to improve the normality of the trait distributions through standard transformations such as log, sqrt, inverse, and asinh, we were unsuccessful. Nonetheless, the residuals of all cases followed the assumption of a normal distribution.

**Figure 1:**
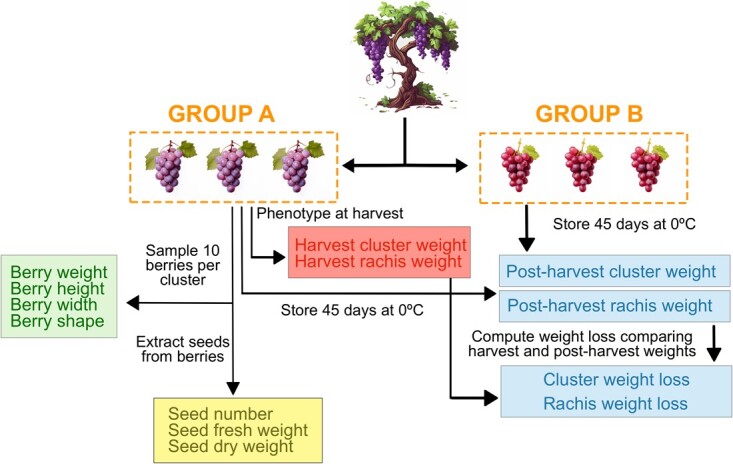
**Schematic workflow of the phenotyping process**. Berry, seed, and harvest cluster/rachis weights were phenotyped at the time of harvest from Group A’s clusters. After 45 days at storage, cluster weight was phenotyped from Group A’s clusters and rachis weight was phenotyped from Group B’s clusters.

**Figure 2:**
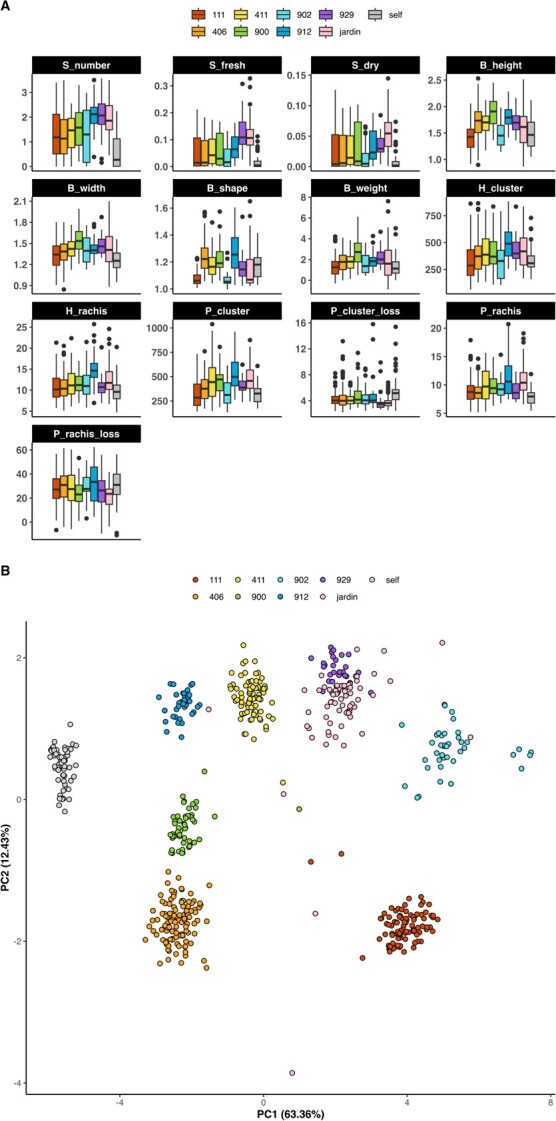
**(A)** Adjusted phenotypic values (BLUEs) for each trait with genotypes grouped by families, also denoted with color. **(B)** PCA based on SNP markers. S_number: number of seeds, S_fresh: seed fresh weight, S_dry: seed dry weight, B_height: berry height, B_width: berry width, B_shape: berry shape (height-to-width ratio), B_weight: berry weight, H_cluster: cluster weight at harvest, H_rachis: rachis weight at harvest, P_cluster: cluster weight at 45 days post-harvest, P_cluster_loss: percentage of cluster weight loss after 45 days, P_rachis: rachis weight at 45 days post-harvest, P_rachis_loss: percentage of rachis weight loss after 45 days.

### Population structure, genetic relatedness, and linkage equilibrium decay

**Figure 3:**
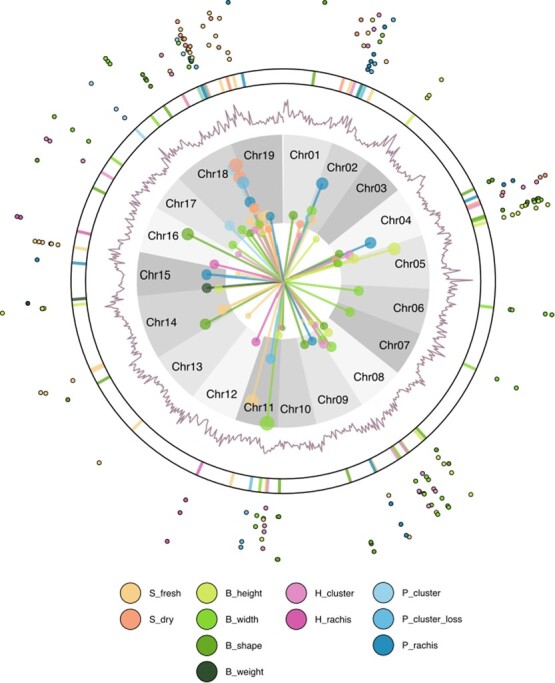
**Multi-layered summary of GWAS**. Colors indicate the different traits, and significant associations are represented in the inner Manhattan plot. The length of the line and the size of the point are proportional to the LOD value. The white circle’s radius represents the Bonferroni threshold (LOD > 5.99), where points in the gray area indicate associations above this threshold. The second layer shows the density of SNP markers. The third layer is a schematic representation of 20-kbp bins in which the gene search was performed. The outermost layer displays the number of genes found in each region (one gene per point). S_fresh: seed fresh weight, S_dry: seed dry weight, B_height: berry height, B_width: berry width, B_shape: berry shape (height-to-width ratio), B_weight: berry weight, H_cluster: cluster weight at harvest, H_rachis: rachis weight at harvest, P_cluster: cluster weight at 45 days post-harvest, P_cluster_loss: percentage of cluster weight loss after 45 days, P_rachis: rachis weight at 45 days post-harvest.

Figure 3B shows the Principal Component Analysis (PCA) output, where the first eight PCs explain 92.27% of the total genetic variance. We detected nine clusters, including the seven crosses, the diversity panel group (*jardin*), and a ninth group of self-pollinated accessions. PC1 explains 63.36% of the total genetic variance and separates accessions based on their female ascendant. The PC1 axis divides the accessions based on their female parent, where the left portion of the axis represents four families of half-sibs (*406, 411, 900*, and *912*) with cultivar 23 (Ruby Seedless × Centennial Seedless) as their female parental line and the self-pollinated family with cultivar 23 being both the female and male parental. The right portion of the axis includes the *jardin* group and the three remaining families (*111, 902*, and *929*). In contrast, PC2, which accounts for 12.43% of the total genetic variance, enables clear identification of all the clusters of full-sibs. The lower portion of PC2 is occupied by two families (111 and 406) that share Crimson (the variety from the diversity panel with the lower value for PC2) as the male parent.

**Figure 4:**
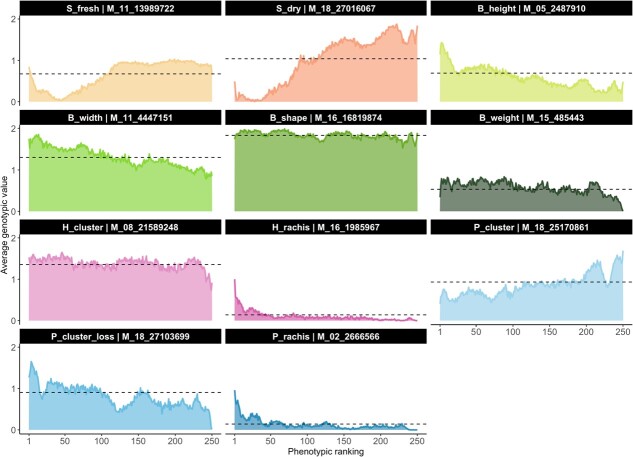
**Marker selection using the most significant SNP as candidates**. The y-axis displays the average genotypic value of the most significant SNP detected for a given trait. The x-axis represents the ranking position, with 1 indicating the highest phenotypic value and the 250 indicating the lowest phenotypic value for a particular trait. For each ranking position, the mean genotypic value is determined by averaging 200 replications of a procedure that randomly samples a subset of 250 accessions, sorts them based on their phenotypic value, and assigns the genotypic value to the ranking positions. The dashed line represents the mean genotypic value discovered in the entire population for the SNP of interest. The highest LOD from the GWAS analysis links each trait to the SNP. We measured the following traits: S_fresh: seed fresh weight, S_dry: seed dry weight, B_height: berry height, B_width: berry width, B_shape: berry shape (height-to-width ratio), H_cluster: cluster weight at harvest, H_rachis: rachis weight at harvest, P_cluster: cluster weight at 45 days post-harvest, P_cluster_loss: percentage of cluster weight loss after 45 days, P_rachis: rachis weight at 45 days post-harvest.

According to the kinship and k-means analysis, there are nine distinct clusters, which can be further categorized into two superclusters, which are consistent with the findings of the PCA. The first supercluster includes all the lines with accession *23* as their female parental line, while the second supercluster comprises the remaining lines. Analysis of linkage disequilibrium (LD) decay indicates that an average LD decay occurs at a distance of 10 kbp for a correlation threshold of ${r}^2=0.2$, as shown in Supplementary Fig. S4.

**Figure 5:**
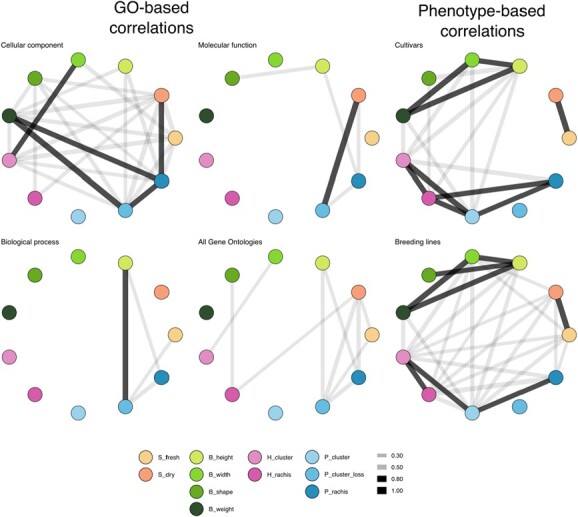
**Correlation between traits**. The Pearson’s r between traits is represented in each graph, with nodes representing each trait. The width of the edges between nodes is proportional to the strength of the correlation, with edges shown in grey for 0.3 < r > 0.7 and highlighted in black for r > 0.7. Correlations were calculated using two methods: GO-based correlations, which used GO frequencies as features, and phenotype-based correlations, which used BLUE values for cultivars (jardin) or breeding lines (other families). We measured the following traits: S_fresh: seed fresh weight, S_dry: seed dry weight, B_height: berry height, B_width: berry width,B_shape: berry shape (height-to-width ratio), B_weight: berry weight, H_cluster: cluster weight at harvest, H_rachis: rachis weight at harvest, P_cluster: cluster weight at 45 days post-harvest, P_cluster_loss: percentage of cluster weight loss after 45 days, P_rachis: rachis weight at 45 days post-harvest.

### Genome-wide association study

We used the BLINK algorithm to assess a panel of 588 genotypes for 13 yield-related traits using a total of 49 210 SNP markers. Our QQ-plots showed deviations from the null hypothesis of no association for 11 of the 13 traits, as detailed in Supplementary Fig. S5. We did not observe any association for *S_number* and *P_rachis_loss*. We identified 69 significant associations (Table 1) above the false discovery rate (FDR) threshold, and 49 of these associations also exceeded the Bonferroni threshold ($\alpha =\frac{0.05}{49210},\mathrm{LOD}>5.99$). Chromosome chr18 had the highest number of associations (16), while chromosomes chr 3, chr 6, chr 7, chr 10, chr 12, and chr 13 had only one association each. Among the 11 traits with significant hits, *B_weight* had the fewest associations (1), while *B_width* (11) and *S_fresh* (14) had the highest number of associations. Additionally, *H_rachis* and *P_cluster* showed two associations each as shown in Fig. 3.3.

**Figure 6:**
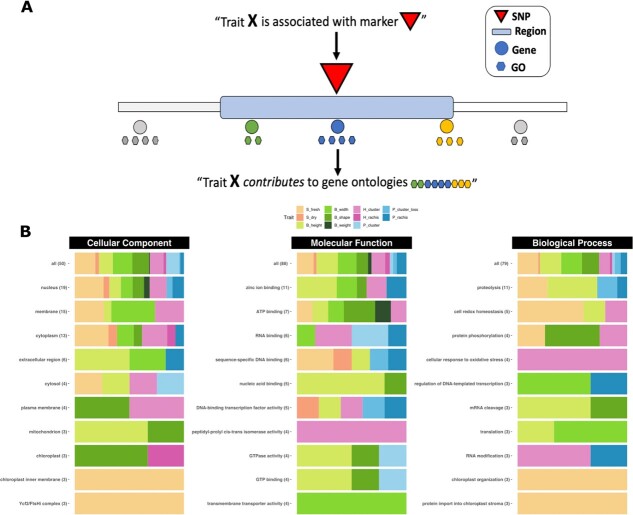
**Contribution of traits to the most frequent GOs**. To link traits to the most frequent GO terms, we followed a process represented in (A) by means of regions and genes. (B) For each type of GO, including CC, MF, and BP, we displayed the total number of GOs and the top 10 most frequent ones in (B). The bars were colored based on the proportion of GO occurrences that were found for each trait, using the pipeline shown in (A). S_fresh: seed fresh weight, S_dry: seed dry weight, B_height: berry height, B_width: berry width, B_shape: berry shape (height-to-width ratio), B_weight: berry weight, H_cluster: cluster weight at harvest, H_rachis: rachis weight at harvest, P_cluster: cluster weight at 45 days post-harvest, P_cluster_loss: percentage of cluster weight loss after 45 days, P_rachis: rachis weight at 45 days post-harvest.

**Table 1:** **GWAS results**. Information about markers associated with traits is presented in the table, including their physical position (chromosome and exact bp) and LOD score. Additionally, we provide annotation information on the closest gene, such as gene name, BLAST similar proteins, and gene ontologies of the protein products of these genes

**Trait**	**Chr**	**Position**	**SNP**	**LOD**	**Region ini**	**Region fin**	**Gene ini**	**Gene fin**	**Gene**	**BLAST hits**	**dist (bp)**	**Gene ontologies**
	1	22 306 525	M_01_22 306 525	5.37	22 281 525	22 331 525	22 304 288	22 306 564	Vitvi01g02270		0	
	2	6 686 801	M_02_6 686 801	6.9	6 661 801	6 711 801	6 682 700	6 682 873	Vitvi02g01487		3928	
	9	7 065 439	M_09_7 065 439	4.93	7 040 439	7 090 439	7 066 313	7 072 358	Vitvi09g00629	XP 002265016.1 adenine phosphoribosyltransferase 5	874	Adenine salvage, AMP salvage, purine ribonucleoside salvage, cytoplasm, adenine phosphoribosyltransferase activity
	11	13 989 722	M_11_13 989 722	14.68	13 939 722	14 039 722						
	13	8 151 231	M_13_8 151 231	5.29	8 126 231	8 176 231	8 148 964	8 153 524	Vitvi13g00739		0	
**S_fresh**	14	6 659 734	M_14_6 659 734	6.84	6 634 734	6 684 734	6 654 655	6 665 536	Vitvi14g00427	XP 002277583.1 pollen-specific protein SF21	0	
		7 345 807	M_14_7 345 807	6.64	7 320 807	7 370 807	7 337 163	7 389 121	Vitvi14g00472	XP 002274609.1 probable inactive ATP-dependent zinc metalloprotease FTSHI 5, chloroplastic	0	Cell redox homeostasis, chloroplast organization, protein import into chloroplast stroma, proteolysis, regulation of photorespiration, chloroplast inner membrane chloroplast thylakoid, Ycf2/FtsHi complex, ATP binding, ATP hydrolysis activity, ATP-dependent peptidase activity, chloroplast protein-transporting ATPase activity, metalloendopeptidase activity
	15	16 996 087	M_15_16 996 087	5.07	16 971 087	17 021 087	16 991 674	16 996 074	Vitvi15g00913	XP 010661482.1 uncharacterized membrane protein At1g16860	13	Membrane
		19 681 084	_M_18 19 681 084	6.89	19 656 084	19 706 084	19 682 424	19 685 819	Vitvi18g01490		1340	
	1	26 994 029	_M_18 26 994 029	5.59	26 969 029	27 019 029	26 991 171	26 994 231	Vitvi18g03065		0	
		27 016 113	_M_18 27 016 113	7.77	26 991 113	27 041 113	27 018 029	27 018 546	Vitvi18g01900		1916	
	19	3 265 960	_M_19 3 265 960	6.84	3 240 960	3 290 960	3 265 452	3 267 338	Vitvi19g00252	XP 002284800.1 CBS domain-containing protein CBSX5	0	Cellular response to glucose starvation, protein phosphorylation, regulation of catalytic activity, cytoplasm, nucleotide-activated protein kinase complex, nucleus, AMP binding, protein kinase binding, protein kinase regulator activity
		5 737 748	_M_19 5 737 748	5.77	5 712 748	5 762 748	5 736 473	5 737 761	Vitvi19g00425	XP 010644401.1 ankyrin repeat-containing protein At5g02620-like	0	
		7 843 732	_M_19 7 843 732	6.91	7 818 732	7 868 732	7 842 956	7 844 371	Vitvi19g00626	RVW50255.1hypothetical protein CK203 116 013	0	Cytoplasm, nucleus
	1	19 586 589	_M_01_19 586 589	6.09	19 561 589	19 611 589	19 584 338	19 585 799	Vitvi01g01459	XP 002263365.1 GEM-like protein 5	790	
	4	19 371 161	_M_04_19 371 161	5.86	19 346 161	19 396 161	19 354 505	19 373 873	Vitvi04g01364	XP 010649104.1PREDICTED: uncharacterized protein LOC100260906	0	
		26 395 984	M_18_26 395 984	12.61	26 370 984	26 420 984	26 391 565	26 392 582	Vitvi18g01868	XP 019071955.1 myb-related protein 308-like	3402	
**S_dry**	18	26 585 996	M_18_26 585 996	6.16	26 560 996	26 610 996	26 580 060	26 581 774	Vitvi18g01875	XP 002272228.1 transcription factor TCP8	4222	Nucleus, DNA-binding transcription factor activity, sequence-specific DNA binding
		27 016 067	M_18_27 016 067	14.96	26 991 067	27 041 067	27 018 029	27 018 546	Vitvi18g01900		1962	
		27 641 922	M_18_27 641 922	7.93	27 616 922	27 666 922	27 645 019	27 647 663	Vitvi18g01937	XP 010665429.1 putative wall-associated receptor kinase-like 16	3097	
	19	5 737 748	M_19_5 737 748	5.66	5 712 748	5 762 748	5 736 473	5 737 761	Vitvi19g00425	XP 010644401.1 ankyrin repeat-containing protein At5g02620-like	0	
	3	3 308 146	M_03_3 308 146	5.62	3 283 146	3 333 146	3 308 692	3 320 395	Vitvi03g00299	RVW48272.1Salicylate carboxymethyltransferase	546	
	5	986 588	M_05_986 588	7.48	961 588	1 011 588	984 657	1 007 813	Vitvi05g00106	XP 010649527.1 bromodomain and WD repeat-containing protein 3 isoform X1	0	Cytoskeleton organization, regulation of cell shape, regulation of transcription by RNA polymerase II, nucleus
		2 487 910	M_05_2 487 910	13.15	2 462 910	2 512 910	2 487 510	2 488 673	Vitvi05g00235	NP 001268197.11-Cys peroxiredoxin	0	Thioredoxin-dependent peroxiredoxin activity, cell redox homeostasis, cytosol, mitochondrion, thioredoxin-dependent peroxiredoxin activity
**B_height**	8	11 609 006	M_08_11 609 006	6.88	11 584 006	11 634 006	11 608 451	11 609 203	Vitvi08g00929	RVW15889.1Aspartic proteinase CDR1	0	
		20 838 429	M_08_20 838 429	5.34	20 813 429	20 863 429	20 837 623	20 841 031	Vitvi08g01794	RVW46980.1IQ domain-containing protein IQM6	0	
	11	2 440 870	M_11_2 440 870	5.69	2 415 870	2 465 870	2 439 052	2 442 344	Vitvi11g00257	XP_010656256.1 protein IQ-DOMAIN 14	0	
	14	28 926 364	M_14_28 926 364	6.59	28 901 364	28 951 364	28 923 196	28 926 504	Vitvi14g01907	XP_002277221.2 probable WRKY transcription factor 72 isoform X1	0	Nucleus, DNA-binding transcription factor activity, sequence-specific DNA binding
	2	2 926 867	M_02_2 926 867	7.63	2 901 867	2 951 867	2 886 776	2 931 532	Vitvi02g00329	XP 010660765.1 lysine-specific demethylase 5B isoform X3	0	Methylation, regulation of DNA-templated transcription, nucleus, DNA binding, metal ion binding, methyltransferase activity
	5	872 428	M_05_872 428	5.99	847 428	897 428	868 859	873 471	Vitvi05g00090		0	
	6	4 874 960	M_06_4 874 960	7.67	4 849 960	4 899 960	4 874 372	4 880 988	Vitvi06g00389	XP 002284532.2 probable NOT transcription complex subunit VIP2 isoform X1	0	Nuclear-transcribed mRNA poly(A) tail shortening, regulation of DNA-templated transcription, CCR4-NOT core complex, P-body
**B_width**	7	4 139 218	M_07_4 139 218	7.39	4 114 218	4 164 218	4 132 329	4 139 353	Vitvi07g00394	XP 002275221.1 dnaJ protein ERDJ3B	0	Protein refolding, cytoplasm, chaperone binding, unfolded protein binding
	8	17 678 111	M_08_17 678 111	8.22	17 653 111	17 703 111	17 676 410	17 678 357	Vitvi08g01496		0	
		20 850 513	M_08_20 850 513	7.12	20 825 513	20 875 513	20 848 483	20 853 959	Vitvi08g02367	XP 002281508.3 40S ribosomal protein S28	0	Maturation of SSU-rRNA, ribosomal small subunit assembly, translation, cytosolic small ribosomal subunit, structural constituent of ribosome
	11	4 447 151	M_11_4 447 151	19.1	4 422 151	4 472 151	4 444 763	4 448 289	Vitvi11g00454	XP 010656420.1 protein NRT1/ PTR FAMILY 6.2 isoform X2	0	Oligopeptide transport, transmembrane transport, membrane, transmembrane transporter activity
	17	6 092 969	M_17_6 092 969	6.32	6 067 969	6 117 969	6 090 734	6 093 282	Vitvi17g00518	XP_002266530.2 protein NUCLEAR FUSION DEFECTIVE 4	0	
		7 568 138	M_18_7 568 138	6.66	7 543 138	7 593 138	7 566 258	7 569 958	Vitvi18g00659	RVW56123.1Protein NPGR1	0	
	18	25 163 895	M_18_25 163 895	5.49	25 138 895	25 188 895	25 162 572	25 162 781	Vitvi18g01775	CBI18290.3unnamed protein product, partial	1114	RNA binding
		26 686 497	M_18_26 686 497	6.33	26 661 497	26 711 497	26 686 202	26 689 010	Vitvi18g01881		0	
	1	9 976 460	M_01_9 976 460	6.72	9 951 460	10 001 460	9 958 225	10 030 522	Vitvi01g00836	XP 034685369.1 protein virilizer homolog	0	Embryo development ending in seed dormancy, response to salt stress, nuclear speck, mRNA methyltransferase activity, RNA N6-methyladenosine methyltransferase complex
	4	14 862 225	M_04_14 862 225	6.33	14 837 225	14 887 225	14 859 361	14 871 185	Vitvi04g01015		0	
**B_shape**	5	336 776	M_05_336 776	5.9	311 776	361 776	331 674	336 883	Vitvi05g00035	XP 002270782.1 acylpyruvase FAHD1, mitochondrial	0	Mitochondrion, acetylpyruvate hydrolase activity
	8	10 520 542	M_08_10 520 542	6.18	10 495 542	10 545 542	10 519 601	10 523 796	Vitvi08g00843	XP 019077023.1 probable serine/threonine-protein kinase WNK5	0	Protein phosphorylation, cytoplasm, ATP binding, protein histidine kinase binding, protein serine/threonine kinase activity
	9	16 162 993	M_09_16 162 993	6.75	16 137 993	16 187 993	16 161 179	16 164 595	Vitvi09g01126	XP 002264393.2 photosynthetic NDH subunit of subcomplex B 1, chloroplastic	0	Photosynthetic electron transport in photosystem I, chloroplast, NAD(P)H dehydrogenase complex (plastoquinone)
	10	16 424 430	M_10_16 424 430	5.14	16 399 430	16 449 430	16 426 963	16 432 120	Vitvi10g01968		2533	
	14	2 453 773	M_14_2 453 773	9.12	2 428 773	2 478 773	2 452 756	2 461 383	Vitvi14g00215	XP 010659619.1PREDICTED: uncharacterized protein LOC100254594 isoform X2	0	Nucleus, transcription corepressor activity
	16	16 819 874	M_16_16 819 874	11.59	16 794 874	16 844 874	16 818 864	16 822 044	Vitvi16g01838	XP 010662562.1 cucumisin	0	
	18	12 709 493	M_18_12 709 493	5.98	12 684 493	12 734 493	12 710 589	12 716 432	Vitvi18g01148	XP 002279471.2 ABC transporter B family member 13 isoform X1	1096	
**B_weight**	15	485 443	M_15_485 443	7.72	460 443	510 443	465 016	502 021	Vitvi15g00014	XP 002275285.1 TATA-binding protein-associated factor BTAF1 isoform X2	0	Nucleus, ATP binding, ATP hydrolysis activity, ATP-dependent activity, acting on DNA, ATP-dependent chromatin remodeler activity, DNA binding, TBP-class protein binding
	2	351 130	M_02_351 130	5.24	326 130	376 130	349 796	356 484	Vitvi02g00042	XP 019081532.1 protein SGT1 homolog isoform X1	0	Chaperone binding
	4	20 111 668	M_04_20 111 668	7.14	20 086 668	20 136 668	20 109 346	20 112 709	Vitvi04g01437	XP 002269631.1 peptidyl-prolyl cis-trans isomerase Pin1	0	Peptidyl-prolyl cis-trans isomerase activity, membrane, cytosol, nucleus
**H_cluster**	8	18 000 198	M_08_18 000 198	5.67	17 975 198	18 025 198	17 999 555	18 004 028	Vitvi08g01523	XP 002283672.1 glucomannan 4-beta-mannosyltransferase 9	0	
		21 589 248	M_08_21 589 248	7.48	21 564 248	21 614 248	21 586 857	21 590 446	Vitvi08g01881	XP 002277767.1 G-type lectin S-receptor-like serine/threonine-protein kinase SD3–1 isoform X1	0	Protein phosphorylation, recognition of pollen, membrane, ATP binding, protein kinase activity
	11	2 000 210	M_11_2 000 210	5.1	1 975 210	2 025 210	1 998 468	2 000 661	Vitvi11g00204	RVW43889.1Pentatricopeptide repeat-containing protein	0	
	17	489 172	M_17_489 172	5.51	464 172	514 172	491 337	493 421	Vitvi17g00052	XP 002280728.1 vacuolar protein 8	2165	
	18	19 715 793	M_18_19 715 793	5.99	19 690 793	19 740 793	19 724 351	19 725 457	Vitvi18g01493		8558	
**H_rachis**	12	5 570 431	M_12_5 570 431	6.71	5 545 431	5 595 431	5 568 686	5 571 047	Vitvi12g00381	XP 002269739.2 5′-adenylylsulfate reductase 3, chloroplastic isoform X2	0	Cysteine biosynthetic process, sulfate assimilation, phosphoadenylyl sulfate reduction by phosphoadenylyl-sulfate reductase (thioredoxin), phosphoadenylyl-sulfate reductase (thioredoxin) activity, catalytic activity 161 985 967 M 161985967 7.131960967 2 010 967 1 984 817 1 986 052 Vitvi16g00127–0
	16	1 985 967	M_16_1 985 967	7.13	1 960 967	2 010 967	1 984 817	1 986 052	Vitvi16g00127		0	
**P_cluster**	18	949 485	M_18_949 485	7.75	924 485	974 485	949 048	961 392	Vitvi18g00099	XP 002270499.1 pre-rRNA-processing protein TSR1 homolog isoform X2	0	Endonucleolytic cleavage of tricistronic rRNA transcript (SSU-rRNA, 5.8S rRNA, LSU-rRNA), maturation of SSU-rRNA from tricistronic rRNA transcript (SSU-rRNA, 5.8S rRNA, LSU-rRNA), nucleolus, preribosome, small subunit precursor, GTP binding, GTPase activity, U3 snoRNA binding
		25 170 861	M_18_25 170 861	14.53	25 145 861	25 195 861	25 162 572	25 162 781	Vitvi18g01775	CBI18290.3unnamed protein product, partial	8080	RNA binding
	2	3 949 944	M_02_3 949 944	6.87	3 924 944	3 974 944	3 943 909	3 950 772	Vitvi02g00420	XP 002282434.1 hydroxymethylglutaryl-CoA syn thase	0	Acetyl-CoA metabolic process, farnesyl diphosphate biosynthetic process, mevalonate pathway, sterol biosynthetic process, hydroxymethylglutaryl-CoA synthase activity,
**P_cluster_loss**	11	7 552 711	M_11_7 552 711	7.92	7 527 711	7 577 711	7 548 641	7 554 321	Vitvi11g00663		0	
	18	27 103 699	M_18_27 103 699	11.59	27 078 699	27 128 699	27 090 552	27 102 800	Vitvi18g03067		899	
**P_rachis**	2	2 666 566	M_02_2 666 566	11.38	2 641 566	2 691 566	2 666 209	2 683 214	Vitvi02g00307		0	
	4	17 435 135	M_04_17 435 135	9.96	17 410 135	17 460 135	17 437 440	17 445 220	Vitvi04g01187		2305	
	9	7 543 821	M_09_7 543 821	6.72	7 518 821	7 568 821	7 531 387	7 545 793	Vitvi09g00653	XP 002263706.2PREDICTED: uncharacterized protein LOC100250971	0	Catalytic activity
	15	12 041 437	M_15_12 041 437	7.75	12 016 437	12 066 437	12 041 338	12 042 616	Vitvi15g00557	RVW57747.1Phospholipase A(1) DAD1, chloroplastic	0	Lipid metabolic process, phospholipase activity
	18	26 395 984	M_18_26 395 984	8.73	26 370 984	26 420 984	26 391 565	26 392 582	Vitvi18g01868	XP 019071955.1 myb-related protein 308-like	3402	
	19	10 748 969	M_19_10 748 969	6.69	10 723 969	10 773 969	10 747 971	10 749 212	Vitvi19g00932	XP 002265645.1 scarecrow-like protein 18	0	Meristem initiation, regulation of DNA-templated transcription, nucleus, DNA-binding transcription factor activity, sequence-specific DNA binding

### Associated SNPs as candidates for marker selection

In evaluating marker–trait associations, any significant SNP marker must exhibit a discernible pattern when we arrange accessions based on their phenotypic values. Consider an extreme case: if an SNP accounts for 100% of the variance, accessions coded as “0” should be first, followed by those genotyped as “1”, and lastly by those marked as “2”. Following this logic, if lines with genotype “2” register higher phenotypic values than those with genotype “0”, a negative Spearman’s correlation emerges. However, when phenotypic values rise in lines with genotype “0” over those with “2”, we observe positive correlations. In instances with ambiguous genotype trends, correlations lean towards zero. Given that the assignments “0” and “2” are arbitrary for any two possible homozygous genotypes of a particular SNP, our focus is on the correlation’s absolute value, rather than its original value.

We obtained Spearman’s correlation ($\rho$) values as described in section 2.7 for the 11 traits with significant associations. Those $\rho$ values ranged from $0.516$ (H_cluster) to $0.941$ (S_dry) (Fig. 4). We observed that related traits exhibited similar patterns, as seen in S_fresh ($0.805$) and S_dry ($0.941$), where accessions carrying allele “0” tended to have higher phenotypic values. However, we also identified an interesting phenomenon in the extreme highest phenotypes of S_fresh, which were occupied by accessions carrying allele “1”. We found that B_height ($0.907$) and B_width ($0.936$) exhibited a continuous and smooth decay, whereas B_weight ($0.713$) exhibited an irregular and less informative decay. B_shape ($0.603$) and H_cluster ($0.516$) show the lower values, and their patterns were close to a uniform distribution in which the average genotypic value for each position is expected to be the average genotypic value in the whole population. Rachis-related traits, H_rachis ($0.848$) and P_rachis ($0.718$), showed a similar pattern, where accessions carrying allele “1” were highly likely to be in the top positions. Finally, P_cluster ($0.894$) and P_cluster_loss ($0.803$) show a solid but noisy decay trend.

To further interpret the $\rho$ values, we conducted a second experiment using 10 randomly selected SNP markers for each trait instead of the most significant one (see Supplementary Fig. S7). The overall average Spearman’s correlation was 0, with a high standard deviation of $0.41$.

### Candidate genes and gene ontology

We detected a total of 69 SNPs significantly associated with the phenotypic traits of interest (Table 1). We found that 48 SNPs were located within a gene, while 20 SNPs were situated within 10 kbp of a candidate gene among these identified SNPs (Table 1). The nearest gene to the remaining SNP (S_fresh - chr11:13989722) was located at a distance of 14 792 bp. Our study identified a set of 172 candidate genes (Fig. 3). On average, each GWAS hit was associated with 2.49 genes, resulting in 163 distinct genes, with 154 genes associated with only one hit, and nine genes associated with two hits. Notably, four genes (Vitvi18g01899, Vitvi18g01900, Vitvi19g00424, and Vitvi19g00425) were shared between S_fresh and S_dry, while two genes (Vitvi08g1794 and Vitvi08g1795) were common to both B_height and B_width. We observed a single gene in 13 hits and a maximum of six for hit *S_fresh* - chr15:16996087 followed by five genes for the associations of *B_height* - chr05:02487910, *B_shape* - chr05:00336776, *B_width* - chr08:17678111, and *P_rachis* - chr02:02666566.

Out of the 172 candidate genes, 83 (48.25%) had at least one Gene Ontology (GO) annotation, resulting in a total of 430 GO terms, with an average of 2.5 ontologies per gene. The GO terms were classified into three main categories: Cellular component (118 GO terms), Molecular function (179 GO terms), and Biological process (133 GO terms). The most frequently annotated GO terms were “GO:0005634 nucleus” (19), “GO:0016020 membrane” (15), and “GO:0005737 cytoplasm” (13) for Cellular component; “GO:0008270 zinc ion binding” (11), “GO:0005524 ATP binding” (7), “GO:0003723 RNA binding” (6), and “GO:0043565 sequence-specific DNA binding” (6) for Molecular function; and “GO:0006508 proteolysis” (11) and “GO:0045454 cell redox homeostasis” (5) for Biological process.

### Correlation between traits

We observed strong phenotypic correlations among traits within the same organ (seed, berry, and cluster/rachis) in our study, except for P_cluster_loss, which did not exhibit any correlation with other traits as depicted in (Fig. 5) Berry traits, except for B_shape, exhibited strong correlations among them. B_shape only is correlated with B_height and this correlation is weak in the diversity panel. Lower correlations were found across organs. Both breeding lines and cultivar panels showed weak and null correlations between seed traits and other traits, respectively.

We observed that correlations based on GO were generally lower than those based on phenotypes, except for the subset of GOs related to the Cellular component (CC) category, which was composed of 10 GOs that appeared more than twice. In contrast, correlations within related traits were significantly lower in CC-GOs compared to phenotypic correlations. Specifically, the strong correlations ($r>0.7$) observed were primarily driven by “GO:0005634 nucleus”, which was found in traits such as B_weight and P_cluster_loss. Higher correlations between B_width and H_rachis, and between S_dry and P_rachis, were driven by “GO:0016020 membrane” and “GO:0005737 cytoplasm”, respectively.

The correlations based on the Molecular Function (MF) and Biological Process (BP) subsets are considerably lower, with only one value exceeding 0.7 for each subset. The MF-GO subset exhibits robust correlations between S_dry and P_cluster_loss, which can be attributed to a comparable pattern in the ontologies “GO:0003700 DNA-binding transcription factor activity” and “GO:0043565 sequence-specific DNA binding”, shared with P_rachis. Moreover, the correlations between B_height and P_rachis share the ontologies “GO:0008270 iron ion binding” and “GO:0004185 serine-type carboxypeptidase activity”, whereas B_height and B_shape have ATP/GTP-related common ontologies. In the case of the BP-GO subset, all correlations with values higher than 0.3 are caused by the ontology “GO:0006508 proteolysis”, in which the four correlated traits (B_height, P_cluster, P_cluster_loss, and S_fresh) are over-represented. S_fresh correlations are lower due to a differential pattern based on chloroplast and photorespiration ontologies.

### Trait contribution to gene ontologies

Results for the most frequent GO terms are shown in Fig. 6B. In considering all GO subsets, we observed consistent relative contributions of each trait, aligning with the proportion of GWAS hits for each trait. However, upon analyzing the ten most frequent GO terms for each subset, we discovered the trait-specificity of each GO. Among them, only three GOs showed more than five traits contributing to them, which are “GO:0005634 nucleus” (with 19 contributions from 9 traits), “GO:0005737 cytoplasm” (with 13 contributions from 7 traits), and “GO:0005524 ATP binding” (with 7 contributions from 6 traits). We did not find any common ontologies such as “GO:0016020 membrane” or “GO:0005524 ATP binding” that included post-harvest traits. B_shape was the only trait found in both the organelles “GO:0005739 mitochondrion” and “GO:0009507 chloroplast”.

With regards to seed traits, there is limited information available for S_dry due to only one of its associated genes having GO annotation. Specifically, a transcription factor from the TCP family has been identified near the major QTL of chr18:26 M. The SNP at chr14:7345807 is responsible for all of the ontologies related to chloroplast structure and function in S_fresh. This SNP is located within the gene Vitvi14g00472, which encodes for the ATP-dependent zinc metalloprotease FTSHI 5.

Regarding berry traits, we identified an SNP located within the gene sequence of Vitvi15g00014 (F6I5H6) as the only match for B_weight (chr15:485443). This gene encodes a TATA-binding protein (TBP) and is associated with ontologies related to nuclear positioning, ATP binding, and chromatin remodeling.

B_width determines “GO:0022857 transmembrane transporter activity” exclusively, which is identified in two distinct hits. The first hit contains a polymorphic nucleotide at chr11:4447151 within the gene structure of Vitvi11g00454. This gene encodes three NRT1/PTR FAMILY 6.2 proteins, The second SNP is located at chr17:6092969, positioned near the gene Vitvi17g00516, which encodes an NFD4-like protein.

The ontologies shared by B_height and B_shape include “GO:0005739 mitochondrion”, “GO:00036764 nucleic acid binding”, “GO:0003924 GTPase activity”, “GO:0005525 GTP binding”, and “GO:0006379 mRNA cleavage”.

Two distinct hits contribute to the “GO:0006508 proteolysis” in B_height. The first hit is located at chr08:11609006 and contains the genes Vitvi08g00928 and Vitvi08g02111, which encode for aspartic proteinase CDR1 proteins. The second hit, located at chr14:28926364, contains the gene Vitvi14g03061, which encodes for a serine carboxypeptidase.

Finally, we found that H_cluster exclusively contributes to the ontology “GO:0003755 peptidyl-prolyl cis-trans isomerase activity” due to an SNP located at chr04:20111668 within the sequence of Vitvi04g01437. Additionally, H_cluster is the only trait in which the ontology “GO:0034599 cellular response to oxidative stress” was identified. Two different hits (chr04:20111668 and chr08:18000198) contain candidate regions that harbor the genes Vitvi08g01521 and Vitvi04g01438, respectively. Vitvi08g01521 encodes type II peroxiredoxin E, while Vitvi04g01438 is associated with peptide methionine sulfoxide reductase A5.

## Discussion

### Trait adjustment

Historically, the QTL for SDI on chromosome 18 has been pinpointed as pivotal for grapevine seed presence [24, 25, 27, 52]. Although serving as a reliable control in calibrating GWAS models, its potential to spur false positives in other traits is acknowledged [4, 53]. Our results showed that despite leveraging best linear unbiased estimators (BLUEs) corrections for berry and harvest traits covariate effects in our analysis, post-harvest traits did not exhibit correlations with seed traits. The use of BLUEs corrections in our study may have contributed to the absence of some common QTL previously reported in the literature. For example, the SDI-co-located QTL for berry weight on chromosome 18 [54] was not detected in our analysis. To further investigate this, we conducted additional analyses using uncorrected BLUEs as the response variable for mixed linear model (MLM) and BLINK GWAS models. We found that the SDI QTL appeared at a Bonferroni level in MLM and at an FDR level in BLINK, as described in Supplementary Fig. S6. These findings suggest that the use of BLUEs corrections in our study may have led to the suppression of some common QTL in grapevine traits, highlighting the importance of carefully considering the choice of the response variable in GWAS analyses [4]. Among our adjusted traits, only berry width aligned with the QTL on chromosome 18 with logarithm of odds (LOD) scores of 5.49 and 6.33. Conversely, most unadjusted post-harvest traits, except for the percentage of rachis weight loss after 45 days, displayed a QTL association on chromosome 18 with LOD scores ranging from 8.73 to 14.53, suggesting an indirect association with seed weight. Further investigation is needed to determine the precise nature of this relationship and whether it can be exploited for grapevine breeding purposes.

### Gene ontologies as trait features

Gene ontologies offer a valuable avenue for functional gene comparisons, especially in expansive data analyses [55, 56]. Our study introduces a pioneering methodology leveraging GO to juxtapose traits, focusing on genes spotlighted in GWAS. Tapping into GO annotations, we probed the nexus between potential genes and target traits. Annotations were ranked from 1 to 5, where ascending scores mirror superior annotation quality, corroborated through empirical evidence or literary sources [55].

This approach provides a starting point for identifying potential trait features and their associated candidate genes, with applications in plant breeding. Moreover, similar approaches have been used in other fields such as human health to explore specific regions of interest [57, 58].

We found that the CC subset GOs had higher correlations between traits due to their non-specific nature, as proteins are required in all cellular components for almost all quantitative traits as denoted in Fig. 5. In addition, the MF and BP GOs had lower correlations, which were expected due to their higher specificity. We suggest that the patterns and insights discovered from the MF–GO and BP–GO correlations are more valuable than those found in CC–GO correlations.

### Seed traits

The widely recognized SDI QTL for seedlessness [24, 25, 27, 52] is evident in both seed fresh weight (2 hits) and seed dry weight (4 hits), detailed in Table 1 and Fig. 3.3 from our analysis. Contrasting with traditional models like MLM, which often detect numerous near-significant SNPs [59, 60], the BLINK algorithm pinpoints significant, proximate SNPs. This arises as BLINK removes linked markers based on an LD threshold of ${r}^2>0.7$, often matching physically associated markers. This concurrence might stem from the major QTL effect, accounting for nearly 70% of the variance [23–25]. A discernible trend exists in the SDI region for seed number, visible in Supplementary Materials (Fig. S5), albeit not reaching statistical significance.

Significant SNPs on chromosome 1 for seed fresh (chr01:22306525) and dry weight (chr01:19586589) might align with the WRKY3 QTL noted by [61]. Similarly, QTL for seed traits in linkage groups 2, 4, and 14 described in [4] may correspond to those in our study. The coincident SNP for both seed traits on chromosome 19 at 5 Mbp (chr19:5737748) could match the NDR1 QTL from [62]. This SNP in our study is a structural variant of the Vitvi19g00425 gene, denoted as an ankyrin repeat-containing protein lacking annotated ontologies.

In the SDI QTL, we observed a few GO annotations for candidate genes, which might have resulted in an under-representation of seed dry weight in the GO-related analysis. However, we identified genes Vitvi18g01868 and Vitvi18g01875 as transcription factors from the TCP and MYB families, respectively.

Regarding the significant presence of allele “1” in the accessions with higher fresh seed weights, we identified that six of the top 10 lines came from family 929, all carrying allele “1”. Within the top 50 accessions, family 929 represented 12 (24%). All of these carried allele “1”. Conversely, of the remaining 38 lines, only eight had allele “1”, with the rest having allele “0” (Supplementary Table S9). Importantly, just two accessions had allele “2”, and both ranked in the last quartile (at positions 411 and 433). The variation in allele frequencies can be attributed to the unique characteristic of family 929, which has Italia as its only seeded parental line.

### Berry traits

In our search for berry weight-related QTL, we failed to identify any stable QTL commonly reported in the literature, such as those in [4]. We only found one QTL in linkage group 15 [28]; however, its genetic location deviated considerably from our physical position. The absence of significant SNPs for berry weight might be due to the corrections we made for seed dry weight, as discussed in section 4.1.

For traits associated with size, we discovered QTL on chromosome 5 linked to berry dimensions—height, width, and shape. These QTL might correspond to one highlighted in an earlier study [63]. The gene closest to the berry height QTL is characterized as a bromodomain protein that is associated with cell shape regulation ontologies [56]. In our search for berry width, we detected a noteworthy SNP on chromosome 11 with an LOD score surpassing 19 and a robust Spearman’s correlation ($\rho$ = 0.936). This SNP, interestingly, resides within Vitvi11g00454, encoding a protein designated D7TC02. This protein, a member of the NRT1/PTR family, plays a role in the transmembrane transport of secondary metabolites in response to jasmonic acid. Furthermore, researchers recognize D7TC02 as the last divergent ortholog (LDO) of the Arabidopsis NRT1 protein [64] (locus AT2G26690https://www.arabidopsis.org/servlets/TairObject?id=32128 type = locus).

Concerning ontologies shared by berry height and shape, we identified GO terms including “mitochondrion”, “nucleic acid binding”, “GTPase activity”, “GTP binding”, and “mRNA cleavage”. Genes like Vitvi05g00104 and Vitvi05g00105, which encode for a zinc finger CCHC transcription factor and the subunit 9A of DNA-directed RNA polymerases II, IV, and V, play a part in some of these ontologies pertaining to berry height. Contrastingly, genes such as Vitvi18g01149 and Vitvi18g02813, which encode for a Rac-like GTP-binding protein RAC1 and the subunit RPA12 of DNA-directed RNA polymerase I, respectively, are pivotal to the ontologies related to berry shape.

### Harvest traits

In this study, we explored the QTL linked to cluster weight and rachis weight in grapevine. Given the highly quantitative nature of these traits, influenced by numerous minor gene contributions, the literature hasn’t reached a consensus on their QTL location. Additionally, population or environmental factors might render these QTL unstable. We delved into an exhaustive literature review, consulting sources like [34, 36, 40, 44–46]. Our findings identified two potential overlaps: one on chromosome 11 from [44] and another on chromosome 17 courtesy of [40], both related to cluster weight. However, our search yielded no overlaps for rachis weight. Nevertheless, our data indicates that of all the traits, only cluster weight influenced the response to oxidative stress ontologies, and this was evident through two distinct QTL on chromosomes 4 and 8.

### Post-harvest traits

In this study, we focused on post-harvest traits, a subject not previously examined in depth in the existing literature. To the best of our understanding, we are the first to investigate the genetic underpinnings of these traits in detail. Consequently, our discoveries shed light on the genetic architecture of post-harvest traits, laying a foundation for subsequent research in this domain. Out of all the traits we studied, only seed number and the percentage of rachis weight loss after 45 days of storage remained elusive in yielding significant results. Given that measuring rachis weight is inherently destructive, we could not compute the rachis weight loss using the identical cluster at both harvest and post-harvest for each genotype and experimental setup. This added an element of noise and uncertainty to our phenotypic value calculations, highlighting the importance of developing non-destructive methods for measuring post-harvest traits in grapevine breeding programs.

Our exploration revealed QTL linked to several post-harvest traits. Notably, we found a QTL that overlaps with the seedlessness QTL SDI on chromosome 18. We discovered a novel QTL on chromosome 18 at 1 Mbp for cluster weight at 45 days post-harvest, which was linked to the gene Vitvi18g00099. This gene manifests as the pre-rna-processing TSR1 protein D7UD95 and the LDO of Arabidopsis TSR1 protein [64, 65] (locus AT1G42440 https://www.arabidopsis.org/servlets/TairObject?accession=locus:2035893), essential in ribosome biogenesis, especially its small subunit. Our results provide novel insights into the genetic basis of post-harvest traits and emphasizing the pivotal role of identified QTL in grapevine breeding schemes. We identified two QTL for the percentage of cluster weight loss post-45 days on chromosomes 2 and 11, associating with genes Vitvi02g00420 and Vitvi11g00663, respectively. The annotation of Vitvi02g00420 designates it as a hydroxymethylglutaryl-CoA synthase with roles in acetyl-CoA metabolic events, inclusive of sterol synthesis [56]. Even though [35] described a berry-cracking QTL on LG 11, our detection probably doesn’t align with it given the spatial disparity.

Assessing rachis weight 45 days post-harvest led us to 6 distinct QTL, with chromosome 18’s QTL aligning with SDI. We found these QTL across chromosomes 2, 4, 9, 15, and 19. Except for chromosome 4, all manifested as structural polymorphisms of genes. The genes from QTL on chromosomes 9 and 15 encode proteins involved in catalytic processes, while genes from QTL on chromosomes 18 and 19 are transcription factors from MYB and SCR families, respectively.

## Conclusion

In this study, we identified many SNP markers that were significantly associated with yield-related grape traits. Interestingly, 70% of them were located within an annotated gene. We discovered a novel QTL on chromosome 11 affecting grapevine berry width linked to Vitvi11g00454, a gene instrumental in managing stress via jasmonic acid, which encodes an NRT1/PTR protein that is recognized as the LDO of Arabidopsis’ NRT1/ PTR FAMILY 6.2 protein. This suggests genetic potential for breeding larger berries. Additionally, we identified QTL influencing post-harvest traits on chromosomes 2, 4, 9, 11, 15, 18, and 19. These findings contribute to the understanding of genetic factors that underlie the fruit’s susceptibility to decay, shriveling, and weight loss after harvest. Furthermore, our results highlight the need to develop non-destructive methodologies that can accurately assess long-term conservation traits. These insights are valuable for grape breeders and growers who seek to improve the post-harvest quality of table grapes and reduce waste. Additionally, our study highlights the importance of carefully considering the choice of the response variable in GWAS analyses, as the use of BLUEs corrections in our study may have led to the suppression of some common QTL in grapevine traits. Overall, our approach of using gene ontology annotations to compare traits and examine candidate genes may provide a useful starting point for identifying potential trait features and their associated candidate genes in plant breeding.

## Material & Methods

### Plant material and experimental design

In this study, we analyzed a total of 68 table grape cultivars (Supplementary Table S1) sourced from the germplasm collection of the Instituto de Investigaciones Agropecuarias (INIA) in Chile and 536 segregating individuals from seven related ${F}_1$ families (Supplementary Table S2) from the INIA table grape breeding program. These families were generated from directed pollination of traditional varieties, including Crimson Seedless (Crimson), Flame Seedless (Flame), and Italia; selections from INIA’s breeding program, such as 23 (Ruby Seedless × Centennial Seedless), 5 (Red Seedless × Dawn Seedless), and Iniagrape-one (Flame Seedless × Black Seedless, also known as Kishmish Chernyi); along with unidentified pollen donors (5LL, 3 V, and 18 V). These crosses were performed in 2010, conducted embryo rescue to obtain all plants, except for those resulting from cross 929, which involved the seeded genotype Italia. We then established single plants at the INIA experimental field in La Platina, La Pintana, Santiago, Chile (33°34’S, 70°37’W, elevation 630 m) in 2013. We planted all vines with row and vine spacing of 3.0 × 1.5 m and trained them using the Guyot system. Drip irrigation was used, and we employed standard agronomic and phytosanitary management practices, except for growth regulator usage, which was not applied.

### Phenotyping

We conducted phenotypic characterization of clusters and berries during the 2018, 2019, and 2020 seasons. At harvest and post-harvest times, we collected six clusters from each ${F}_1$ plant, harvested at 16°Brix, and determined with an analog refractometer on-site. Similarly, we collected six clusters of three plants from each cultivar in the germplasm collection.

For each plant, we divided the six clusters into groups A and B, each consisting of three clusters. We evaluated clusters from group A only at harvest time, measuring nine traits, including cluster weight (g), berry weight (g), soluble solids (°Brix), seed number, seed fresh weight (g), dry seed weight (g), berry height (cm), berry width (cm), berry shape (berry height/berry width), and rachis weight (g). For berry-related traits, we obtained measurements from 10 random berries from each cluster using an in-house script for image analysis. Rachises were weighed after trimming all berries. In contrast, we evaluated harvest and post-harvest parameters of the same clusters from group B, which were labeled, weighed, and packed under standard commercial conditions before being stored at 0°C for 45 days in a controlled atmosphere for future post-harvest evaluations. At post-harvest, we measured four traits: final cluster weight (g), cluster weight loss (percentage), rachis final weight (g), and rachis weight loss (percentage). Weight loss was determined as the difference in weight between harvest and post-harvest, divided by the weight at harvest. We measured the same clusters for cluster weight before and after storage and used different clusters for harvest (from group A) and post-harvest (from group B) evaluation of rachis weight, as it is a destructive measurement. An schematic diagram of the phenotypic process is shown in Figure 1. For the sake of simplicity and clarity, we recorded the original trait names as follows: S_number (number of seeds), S_fresh (seed fresh weight), S_dry (seed dry weight), B_height (berry height), B_width (berry width), B_shape (berry shape, i.e. height-to-width ratio), B_weight (berry weight), H_cluster (cluster weight at harvest), H_rachis (rachis weight at harvest), P_cluster (cluster weight at 45 days post-harvest), P_cluster_loss (percentage of cluster weight loss after 45 days), P_rachis (rachis weight at 45 days post-harvest), and P_rachis_loss (percentage of rachis weight loss after 45 days).

### Genotyping, quality control, and imputation

Genomic DNA from both germplasm collection and breeding families was obtained using a DNAeasy® Plant kit (QiaGen, Germany). Samples from breeding families were extracted once, while samples from the germplasm collection were extracted in duplicate. Sequencing and genotyping were performed at the bioinformatics facility of the University of Minnesota. Samples were processed for genotyping-by-sequencing (GBS) using an ApeK1 enzyme and following standard procedures [66–68]. Pooled samples were sequenced using Illumina HiSeq 2500 equipment. Sequencing reads from each sample were mapped against the *V. vinifera* reference genome PN40024.12X available from Ensembl genomes, using the Bowtie 2 aligner [69] and FreeBayes Software [70] to perform the SNP calling considering diploid. To filter the raw SNP set, we utilized the vcftools software [71], removing non-biallelic sites and those with a minimum allele frequency (MAF) <5%. We also excluded samples with a call rate of <50%. After filtering, we obtained an SNP matrix with 49 210 markers. Missing values were imputed using Beagle 5.4 software [72].

### Population structure, cryptic relatedness, and LD decay

We utilized the set of SNPs obtained after conducting quality control to examine the population structure of the grape cohort through PCA via the *prcomp* function. To validate the PCA analysis, we employed two unsupervised clustering machine learning approaches, namely, the hierarchical (*hclust* function) and the K-means algorithms (*kmeans*). We chose a value of $K=9$ for both methods, as recommended by breeders, to account for a group of cultivars, seven crosses resulting in different families, and a potential group of accidental self-pollinated lines. We evaluated the cryptic genetic relatedness between individuals by computing VanRaden’s kinship matrix using the *AGHmatrix* package [73, 74]. Linkage disequilibrium decay was assessed by calculating the relationship between pairwise squared correlation (${r}^2$) of SNPs and physical distance within 500 Kbp via the *snprelate* package [75].

### Modelling of raw phenotypic data

We performed an adjustment of the phenotypic records prior to conducting GWAS analysis. Specifically, we conducted a linear fixed effects model using *lm* function in base R [76] to remove seasonal and trait-specific covariate effects and obtain the total genetic value via BLUEs. The general model can be expressed as follows:

(1) \begin{equation*} \left\{\begin{array}{l}y={X}_1\beta +{X}_2\omega +{X}_3g+\epsilon \\ {}\epsilon \sim N\left(0,{\sigma}_{\epsilon}^2\right)\end{array}\right. \end{equation*}

where $y$ represents a vector of phenotypic records for a given trait. We employed the fixed effects design matrices ${X}_1$, ${X}_2$, and ${X}_3$. The vector $\beta$ denotes the estimates for seasonal effects, while the vector $\omega$ represents the estimates for trait-specific covariates. The vector $g$ contains the estimates for the total genetic value, and $\epsilon$ is a random and homoscedastic error term.

We selected trait-specific covariates based on the breeder’s knowledge. For berry traits (B_height, B_width, B_shape, B_weight) and harvest traits (H_cluster, H_rachis), we corrected for both soluble solids and seed dry weight (trait S_dry). For post-harvest traits (P_cluster, P_cluster_loss, P_rachis, P_rachis_loss), we corrected using soluble solids. No covariate was used to correct for seed traits (S_number, S_fresh, S_dry).

### Genome-wide association study

We conducted GWAS analysis to evaluate associations between SNPs and 13 yield-related traits using BLUEs as response variable, as described in the preceding section. We utilized the *BLINK* algorithm [59], implemented in R [76] and included in the *GAPIT3* package [60], and incorporated the first six PCs to account for population structure [53]. To reduce type I errors, we adjusted *P*-values using standard FDR and Bonferroni corrections. We evaluated deviations from the null hypothesis of no association between SNPs and traits using Q-Q (Quantile-Quantile) plots, a critical step in detecting confounding factors that could inflate *P*-values [77].

We used the BLINK algorithm because it has been shown to outperform its predecessors, including the general linear model, MLM, and Fixed and random model Circulating Probability Unification (FarmCPU), in terms of both computational efficiency and statistical power, as reported in previous studies [59]. The computational efficiency is achieved by substituting the expensive random effects model, which accounts for genetic relatedness and uses the REML algorithm, with an efficient fixed effect model that is fitted by optimizing the Bayesian information criterion (BIC). Additionally, better control of false positives and false negatives is achieved by overcoming the assumption of uniform quantitative trait nucleotide distribution across the genome. This is done by replacing the bin approach of FarmCPU with an LD-based criterion.

### Evaluation of associated SNPs as candidates for marker selection

When dealing with simple, qualitative traits, the genetic architecture typically revolves around one or few genes. If we have a genomic marker linked to these genes, we will be able to i) precisely select genotypes that are likely to express the desired phenotype and ii) identify statistically significant phenotypic distinctions by categorizing genotypes according to the alleles of said marker or markers.

However, when it comes with complex, quantitative traits governed by numerous genes, the pursuit of markers that lead to significant phenotypic disparities in populations exhibiting alternative alleles becomes more challenging. The complexity arises from the fact that even if we find a statistical causative link through GWAS, the markers’ capacity to account for phenotypic variance may remain relatively limited.

To assess whether a marker contributes to differences, a strategy involves arranging the genotypes based on their phenotypic records. Subsequently, an assessment is made of both the local and global patterns of allelic frequency. We aim to expand this approach by applying the concept of ‘bagging’ [51], which consists in generating multiple populations or ‘bootstrapped samples’ from the original one by permutation.

To minimize sampling bias, we generate 200 bootstrapped populations by randomly selecting 250 accessions from the pool with replacement , and then we assigned a ranking position based on their phenotypic values (with the accession with the highest phenotypic being ranked as 1 and the accession with the lowest value being ranked as 250). We then averaged the genotypic value found for each of the ranking positions in the 200 bootstrapped populations. We assessed trends using both visual inspection and an analytical method, using Spearman’s $\rho$ correlation between the ranking positions and the average genotypic value. We described the procedure using pseudocode notation in Algorithm 2.7.

**Algorithm** 1. An algorithm for estimating the suitability of a single SNP marker to phenotypically differentiate bootstrapped populations.

**Input**: Vector of accessions ($L$), vector of phenotypic values ($Y$), and vector of genotypic values ($X$).

set $R$ as the number of replicates.

set $S$ as the sample size

initialize an empty matrix $M$ of $R$ rows and $S$ columns

$ \mathbf{for}\ r=1\ \mathrm{to}\ R\ \mathbf{do} $

generate a subset $C$ by randomly sampling $S$ accessions from pool $L.$

reorder $C$ subset based on phenotypic values on $Y$

$ \mathbf{for}\ s=1\ \mathrm{to}\ S\ \mathbf{do} $

set $c$ as the $s$-th line in $C$

$ c\leftarrow {C}_s $

set current element in $M$ as the genotypic value of accession $c$

$ {M}_{rs}\leftarrow {X}_c $

**end for**

**end for**

Compute the average genotypic value of each column in $M$

$ \textrm{output}_s\leftarrow \frac{\sum_{r=1}^R{M}_{rs}}{R} $

**Output:** Vector with the average genotypic value of ranked positions based on phenotype

### Gene annotation and gene ontologies

We used Grapedia as the source for gene annotations of the PN40024.12X gff file in this study. The “gene” rows (column 3) were the only ones kept in the analysis, as the exon/intron structure of each gene was considered irrelevant to our research objectives. Additionally, we converted a table of GWAS hits into bed format, with the first column representing chromosome number and the second and third columns indicating the start and end positions of the hit, respectively. The fourth column retained relevant metadata. The start and end positions were extended to include a window of 25 kbp around each significant SNP. Next, we utilized bedtools intersect [78] to generate a list of genes that intersected with the 25-kbp range around each GWAS hit, using the reduced gff file. Finally, we used Blast2GO (https://www.blast2go.com/) to obtain functional annotations for each gene identifier to provide additional context.

We used the physical positions of markers significantly associated with traits as a reference to identify potential candidate genes. To do this, we selected a bin of 20 kbp around the marker position, which corresponds to $\pm$10 kbp from the SNP position, based on the LD decay pattern of the panel. The variation in LD patterns across chromosomes may reflect complex historical recombination events or selection pressures specific to some genomic regions. Nevertheless, the $\pm$10-kb bin was selected as a conservative threshold to avoid false positives (the detection of genes that are not truly related to significant SNPs). We then searched for all genes located within this bin as candidate genes. In cases where there were no genes in the 20-kbp bin, we extended the search to a larger bin of 50 kbp ($\pm$25 kbp from the SNP position) to identify the closest gene. If the closest gene was located outside the 50-kbp bin, we considered that the hit was missing a candidate gene.

We utilized an *in-house* script to automatically extract GO information from the Uniprot database [56, 79] for all candidate genes within the initial 20-kbp bin, using the *GET* function from the *httr* package [80]. We classified the GOs into three main subcategories: cellular location, molecular function, and biological process, and linked them to traits via SNP associations to generate a trait feature matrix. Only GOs that appeared more than twice were taken into account. We compared pairwise GO-based trait correlations with correlations obtained from phenotypic records and investigated the relative contribution of each trait to the most frequent GOs found in this study, as shown in Fig. 6A. We conducted all analyses using R version 4.2.2 and utilized packages from the *tidyverse* family [81] for data processing, including *dplyr* [82], and used *ggplot2* for visualization [83].

## Supplementary Material

Web_Material_uhad283

## Data Availability

The data underlying the findings presented in this article are available in the GitHub repository maintained by TheRocinante-lab at https://github.com/TheRocinante-lab/Publications/tree/main/2024/GarciaAbadilloEtAl_Dissecting. This repository contains all the data necessary to reproduce the results presented in this paper. Additionally, the repository is freely accessible to anyone who wishes to explore the data or use it for their own research purposes. We encourage interested researchers to take advantage of this resource and to contact us if they have any questions or would like more information about the data.
