# Correction to: Dissecting the complex genetic basis of pre- and post-harvest traits in *Vitis vinifera L.* using genome-wide association studies

**DOI:** 10.1093/hr/uhae091

**Published:** 2024-04-13

This is a correction to: Julian García-Abadillo, Paolo Barba, Tiago Carvalho, Viviana Sosa-Zuñiga, Roberto Lozano, Humberto Fanelli Carvalho, Miguel Garcia-Rojas, Erika Salazar, Julio Isidro y Sánchez, Dissecting the complex genetic basis of pre- and post-harvest traits in *Vitis vinifera L.* using genome-wide association studies, Horticulture Research, Volume 11, Issue 2, February 2024, uhad283, https://doi.org/10.1093/hr/uhad283.

 In the first sentence of the section `Phenotype analysis' the reference to figure 3.1A has been corrected to read 2A.

In the first sentence of the section `Population structure, genetic relatedness, and linkage equlibrium decay' the reference to figure 3B has been corrected to read 2B.

In the last sentence of the section `Genome-wide association study', the reference to figure 3.3 has been corrected to read 3.

In the first sentence of the section `Seed traits', the reference to figure 3.3 has been corrected to read 3.

