# Supplementary material for: Dissecting the complex genetic basis of pre- and post-harvest traits in Vitis vinifera L. using genome-wide association studies
Source: Hortic Res. 2024 Jan 3;11(2):uhad283. doi: 10.1093/hr/uhad283 (PMC10939405; doi:10.1093/hr/uhad283)
Supplement: Web_Material_uhad283 [file web_material_uhad283.zip › readme.rtf]

- Columns ‘segregantes’ for breeding lines and ‘varieties’ for the diversity panel denote the genotype name in the phenotype file.- To match phenotype with genomic files (markers and pedigree), it is required to add the string ‘G_’ to the genotype names in the phenotype file	Example: Genotype name ‘5.2.77’ in phenotype file matches with genotype ‘G_5.2.77’ in the genomic files.
