# Supplementary material for: Dissecting the complex genetic basis of pre- and post-harvest traits in Vitis vinifera L. using genome-wide association studies
Source: Hortic Res. 2024 Jan 3;11(2):uhad283. doi: 10.1093/hr/uhad283 (PMC10939405; doi:10.1093/hr/uhad283)
Supplement: Web_Material_uhad283 [file web_material_uhad283.zip › SUPP_S3_S8.docx]

| **Supplementary table S3.** The variation between families means for all the studied traits was evaluated using a one-way ANOVA. The p-values are presented as the log of odds (LOD). Significance levels were set as p-value > 0.05 (not significant, ns), p-value < 0.05 (*), p-value < 0.01 (**) and p-value < 0.001 (***).. Significant differences for Tukey's honest significant difference (HSD) at a 5% level of significance are denoted by different letters. | | | | | | | | | | | | |
| --- | --- | --- | --- | --- | --- | --- | --- | --- | --- | --- | --- | --- |
|  |  |  |  |  |  |  |  |  |  |  |  |  |

|  |  |  |  |  |  |  |  |  |  |  |  |  |  |  |  |
| --- | --- | --- | --- | --- | --- | --- | --- | --- | --- | --- | --- | --- | --- | --- | --- |
|  | **Trait** |  | **ANOVA** | |  | **POST-HOC TUKEY HSD TEST** | | | | | | | | |  |
|  |  |  | **LOD** | **sign.** |  | **111** | **406** | **411** | **900** | **902** | **912** | **929** | **jar.** | **self** |  |
|  | S_number |  | 23.33 | *** |  | a | a | a | ab | a | c | bc | bc | d |  |
|  | S_fresh |  | 24.43 | *** |  | a | ab | a | a | ab | a | c | c | b |  |
|  | S_dry |  | 14.5 | *** |  | ab | ab | b | b | ab | b | bc | c | a |  |
|  | B_weight |  | 11.78 | *** |  | a | bc | ac | d | ac | ac | cd | ac | ab |  |
|  | B_height |  | 28.32 | *** |  | a | bc | bc | d | ae | bd | cd | ce | ae |  |
|  | B_width |  | 15.71 | *** |  | ab | bc | c | d | bc | bc | cd | c | a |  |
|  | B_shape |  | 38.47 | *** |  | a | b | cd | bc | a | b | cd | d | cd |  |
|  | H_cluster |  | 10.3 | *** |  | a | ab | bc | bde | ace | d | bde | bd | ae |  |
|  | H_rachis |  | 14.46 | *** |  | ab | ab | ac | ac | bc | d | bc | c | b |  |
|  | P_cluster_weight | | 13.51 | *** |  | a | ab | bc | bc | a | c | ac | c | a |  |
|  | P_cluster_loss |  | 5.92 | *** |  | ab | ab | ab | ac | bc | bc | ab | b | c |  |
|  | P_rachis_weight |  | 13.46 | *** |  | ab | bc | ad | acd | abe | d | ab | de | b |  |
|  | P_rachis_loss |  | 3.17 | *** |  | ab | b | ab | ab | ab | b | ab | a | b |  |
|  |  |  |  |  |  |  |  |  |  |  |  |  |  |  |  |

**
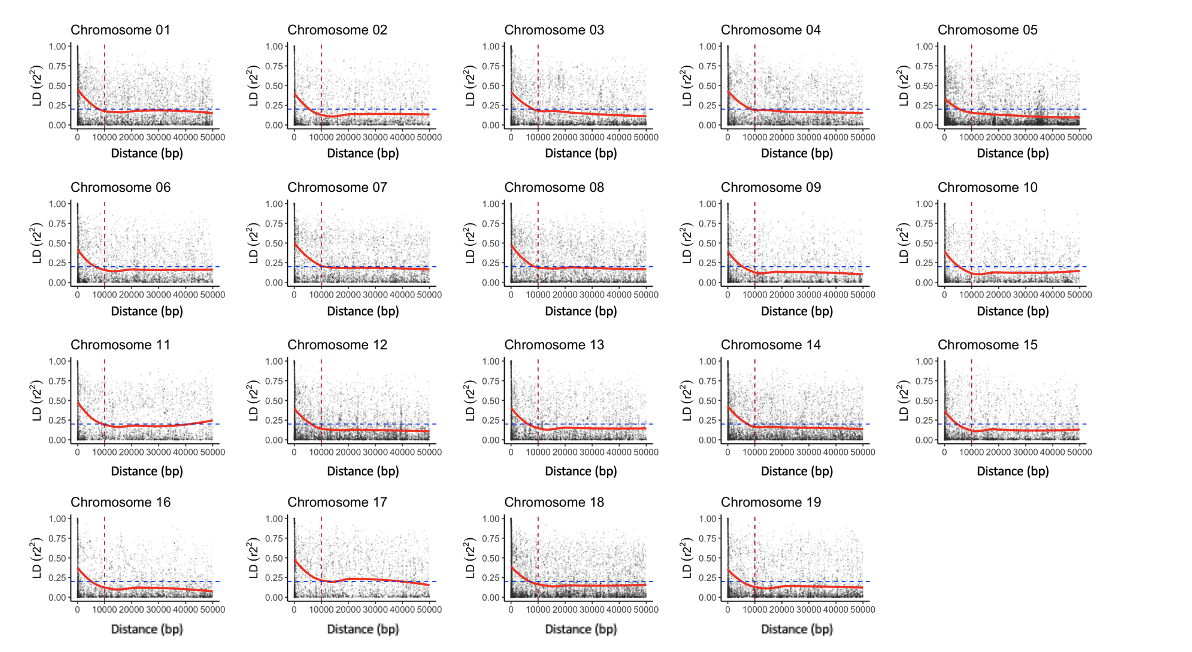
**

**Supplementary figure S4.** This figure illustrates the LD (linkage disequilibrium) decay patterns observed in the subject population for the 19 chromosomes of the grapevine genome. The X-axis shows the pairwise physical distance between markers, which were measured in base pairs (bp), while the Y-axis represents pairwise LD as the squared Pearson's correlation coefficient. The red curve represents the average LD for a given distance. A physical distance of 10,000 bp is denoted by a vertical dashed red line, and a horizontal dashed blue line represents a squared Pearson's correlation coefficient of 0.2.distance. A physical distance of 10,000 bp is denoted by a vertical dashed red line, and a horizontal dashed blue line represents a squared Pearson's correlation coefficient of 0.2.


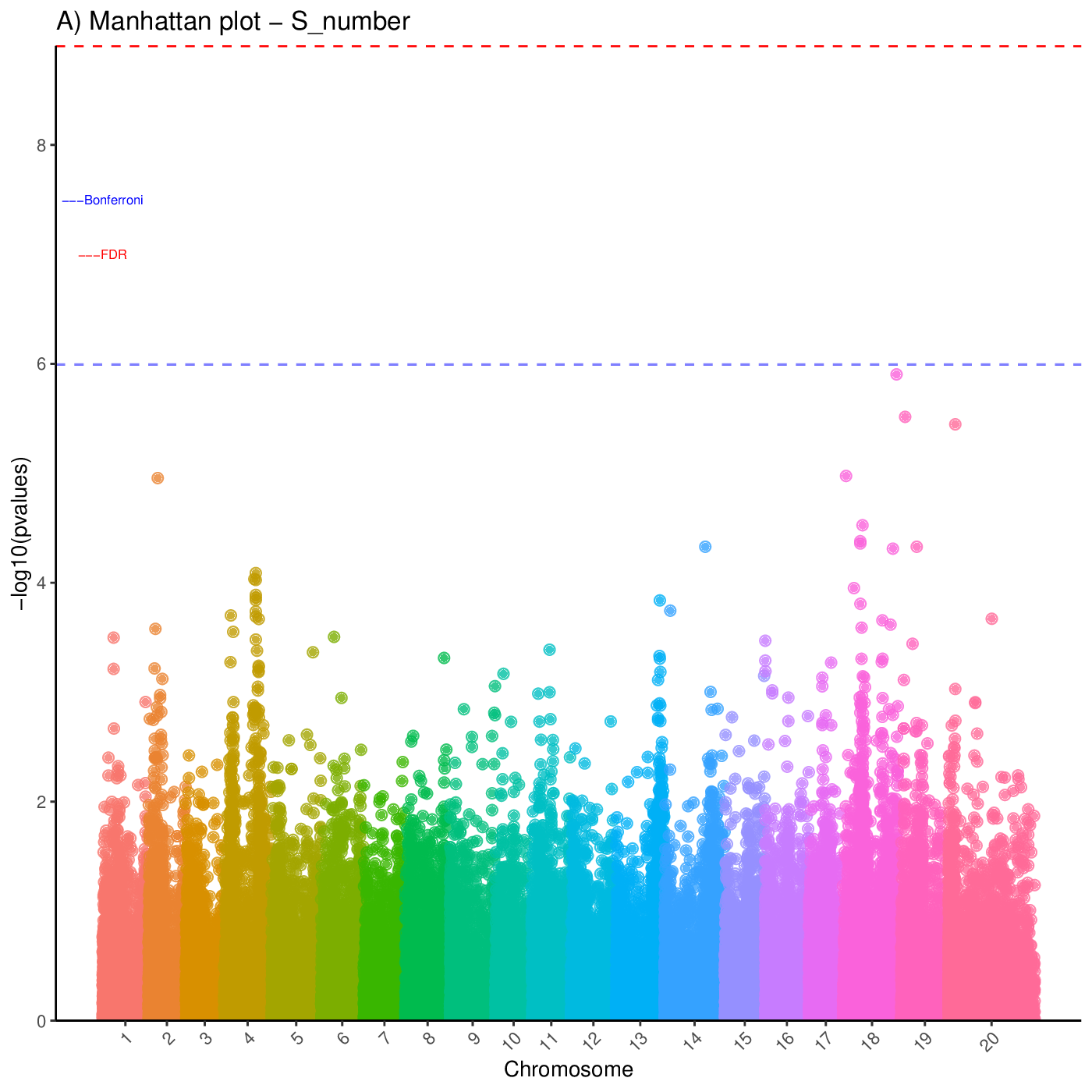

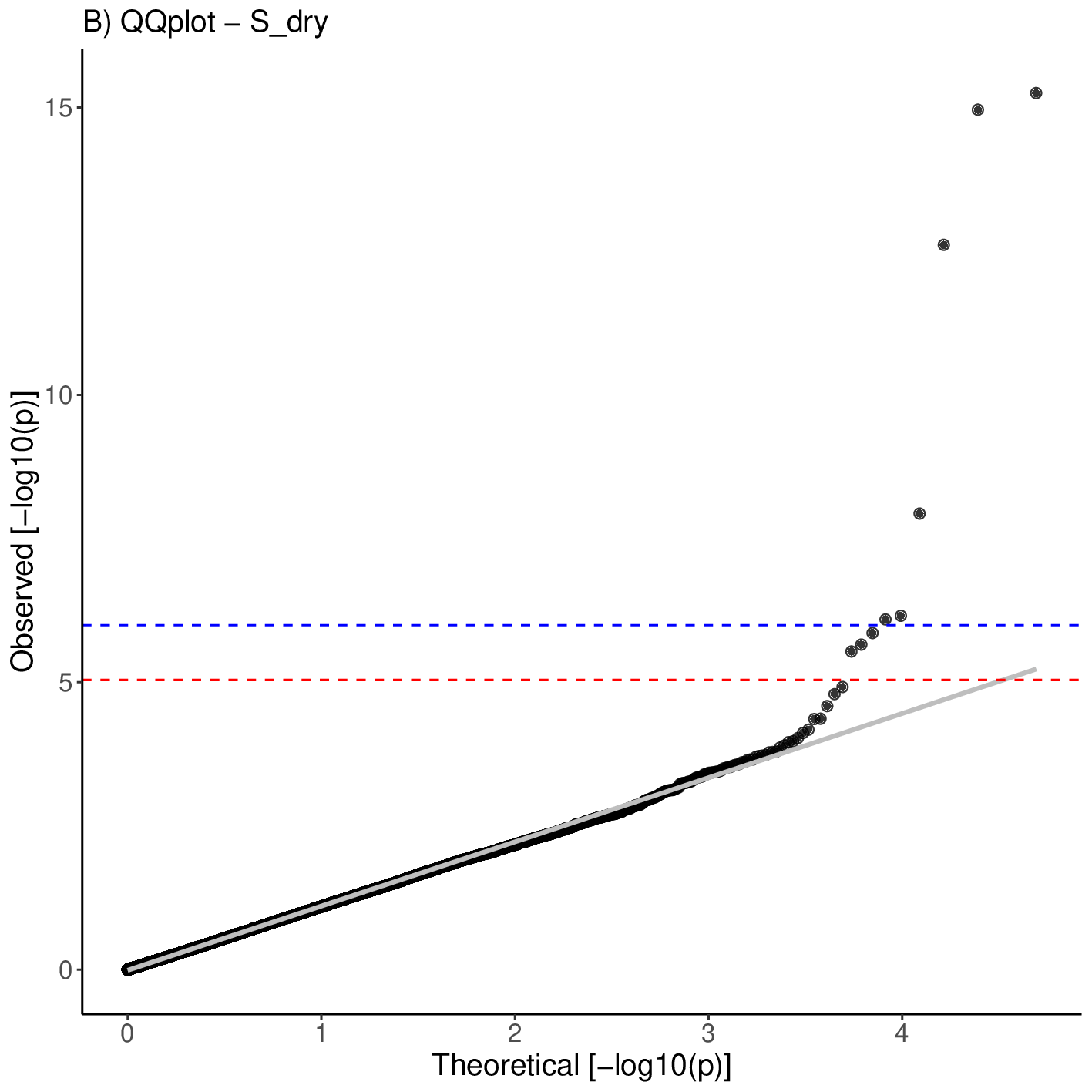

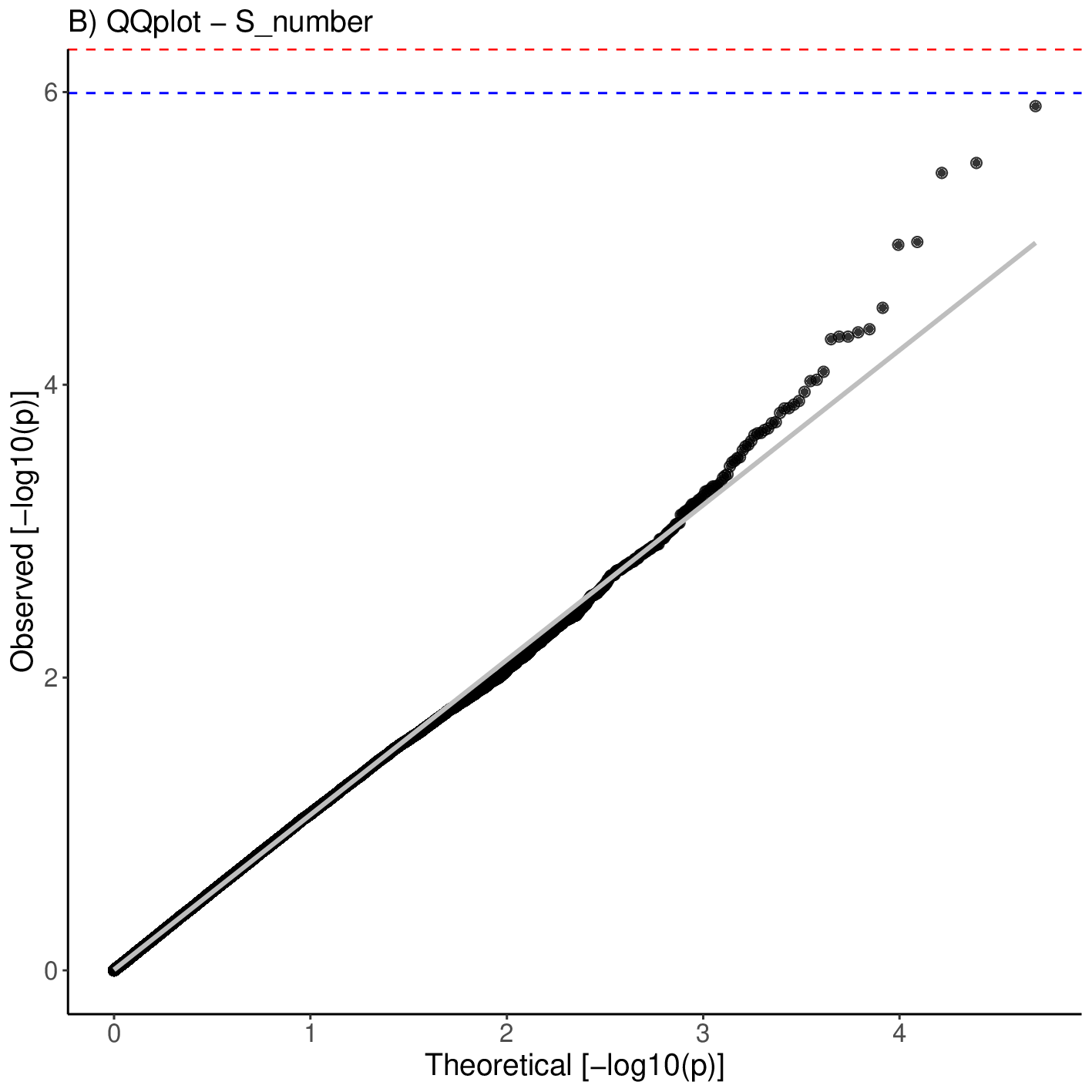

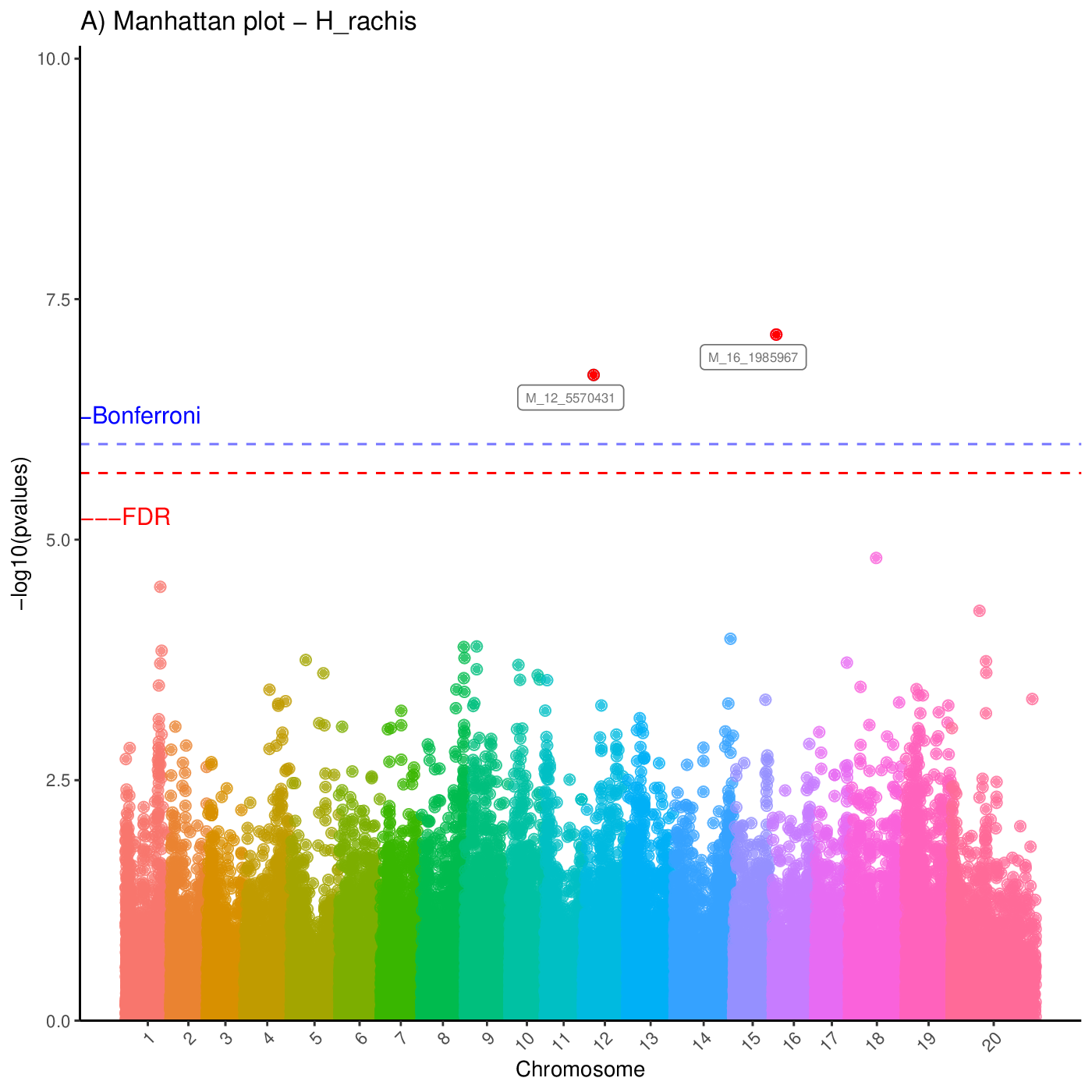

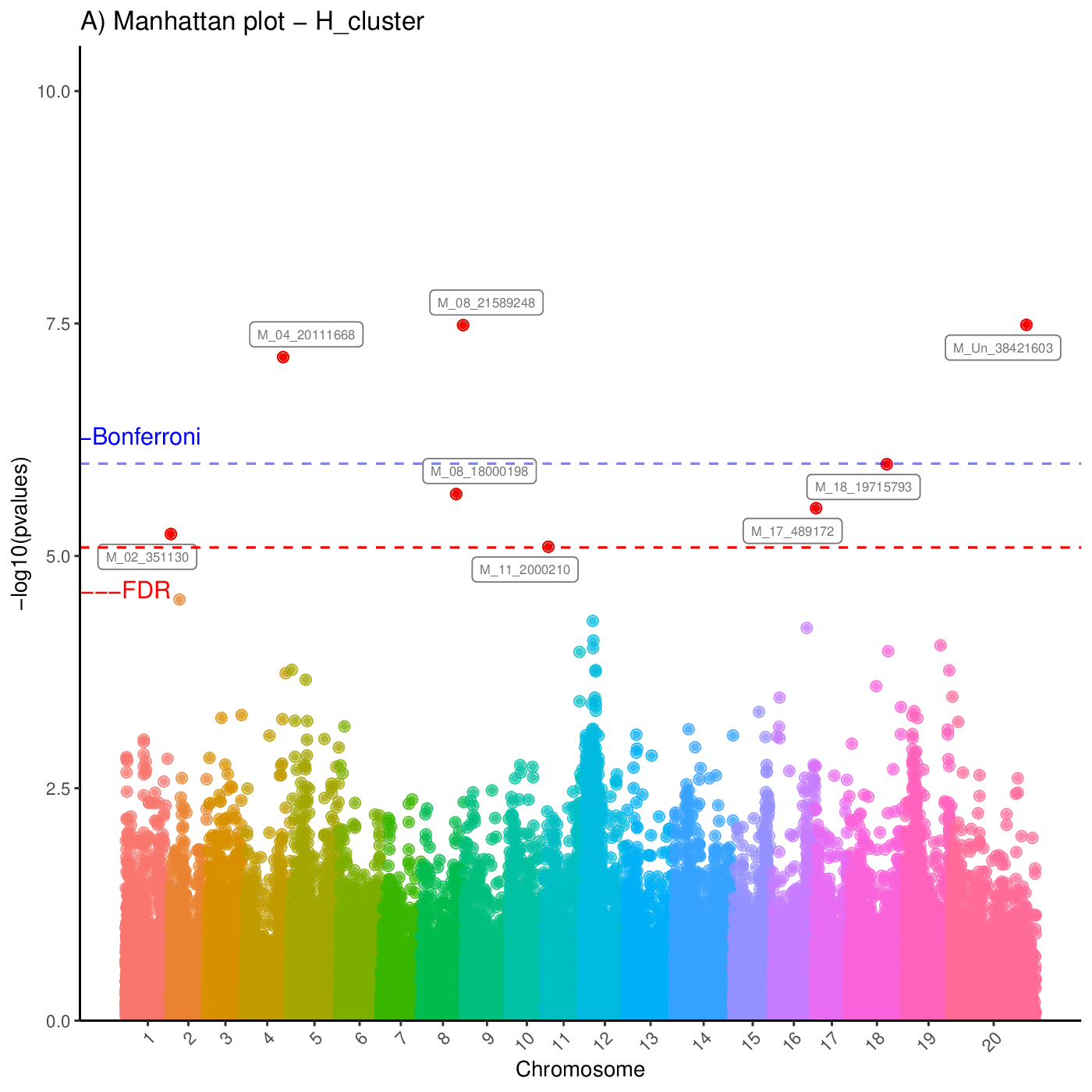

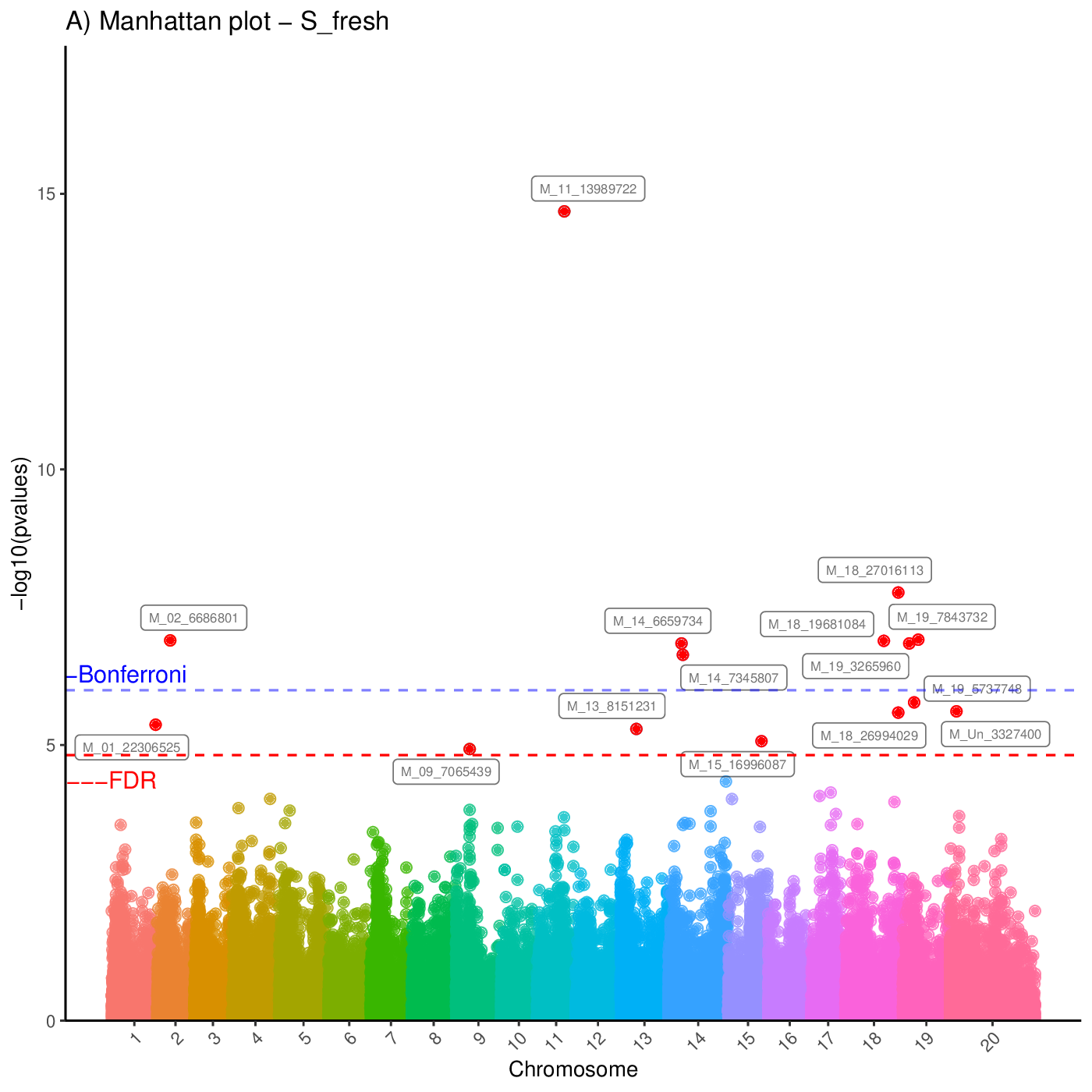

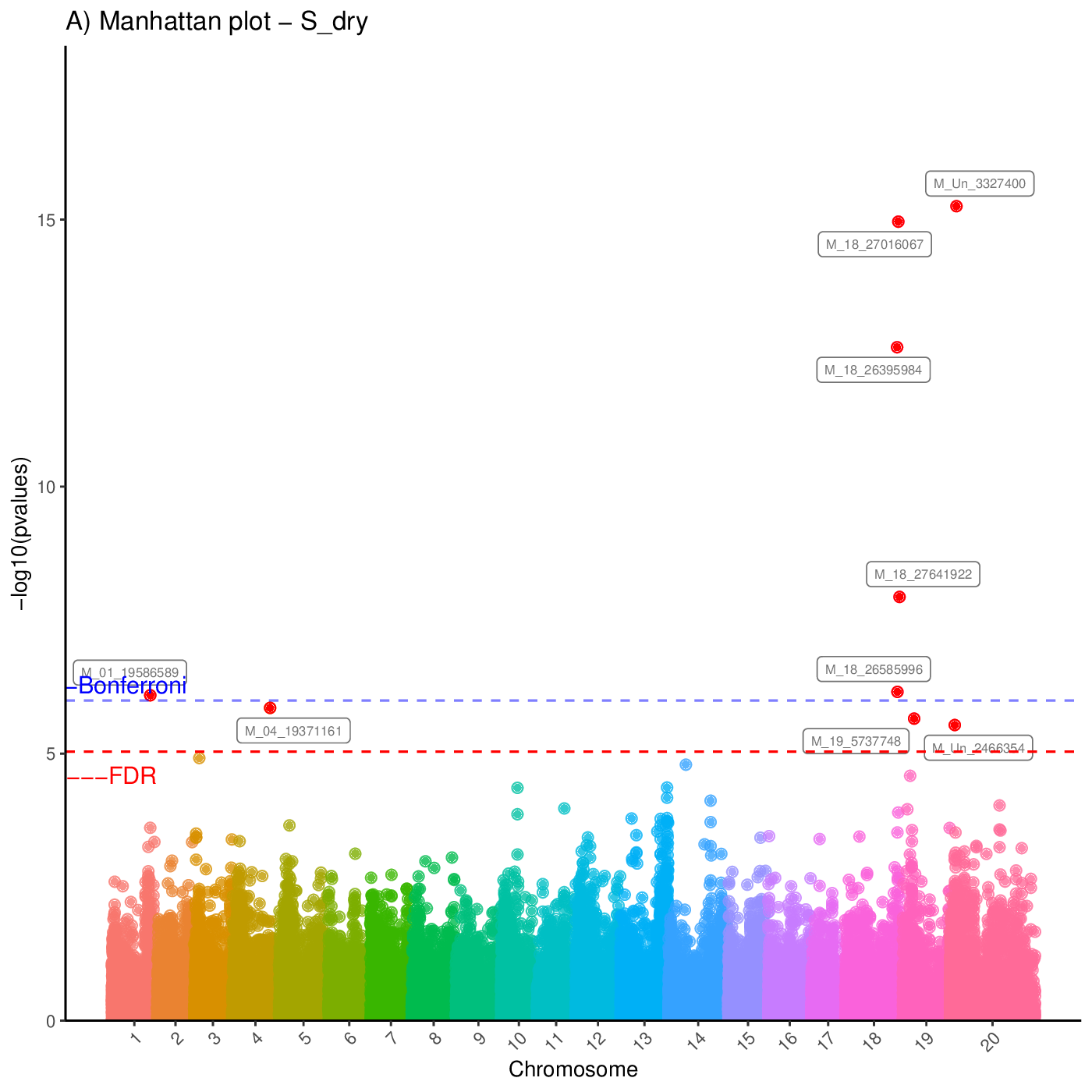

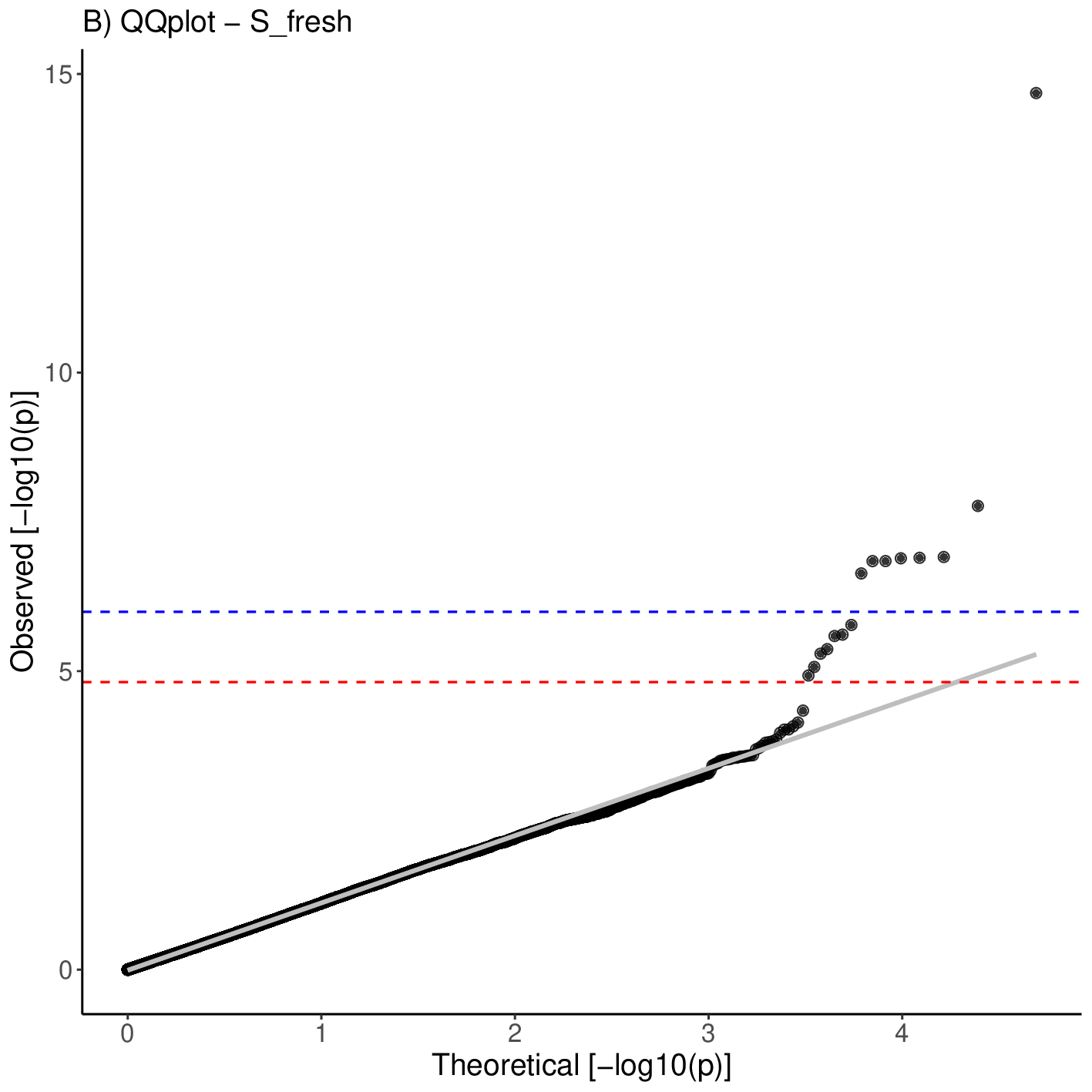

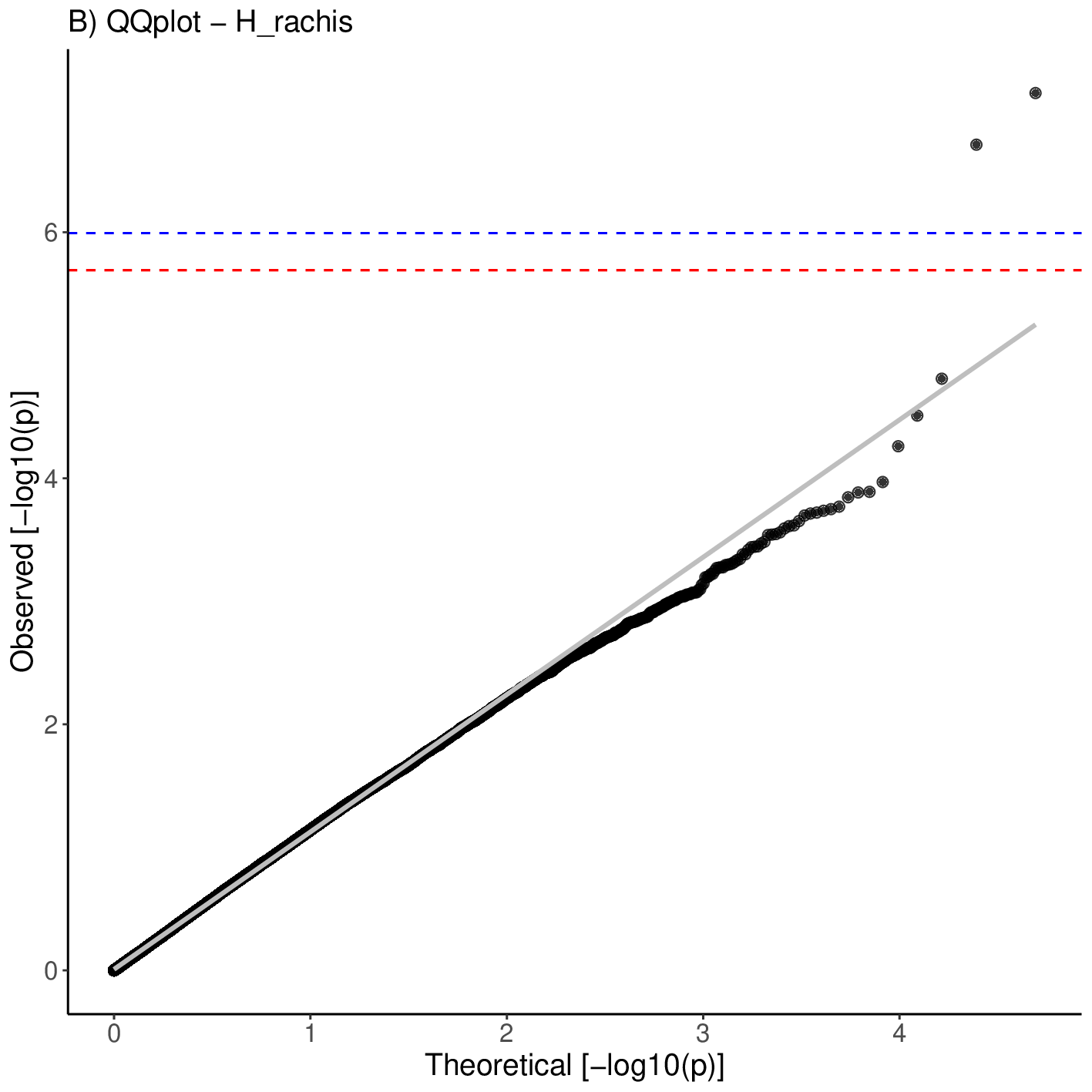

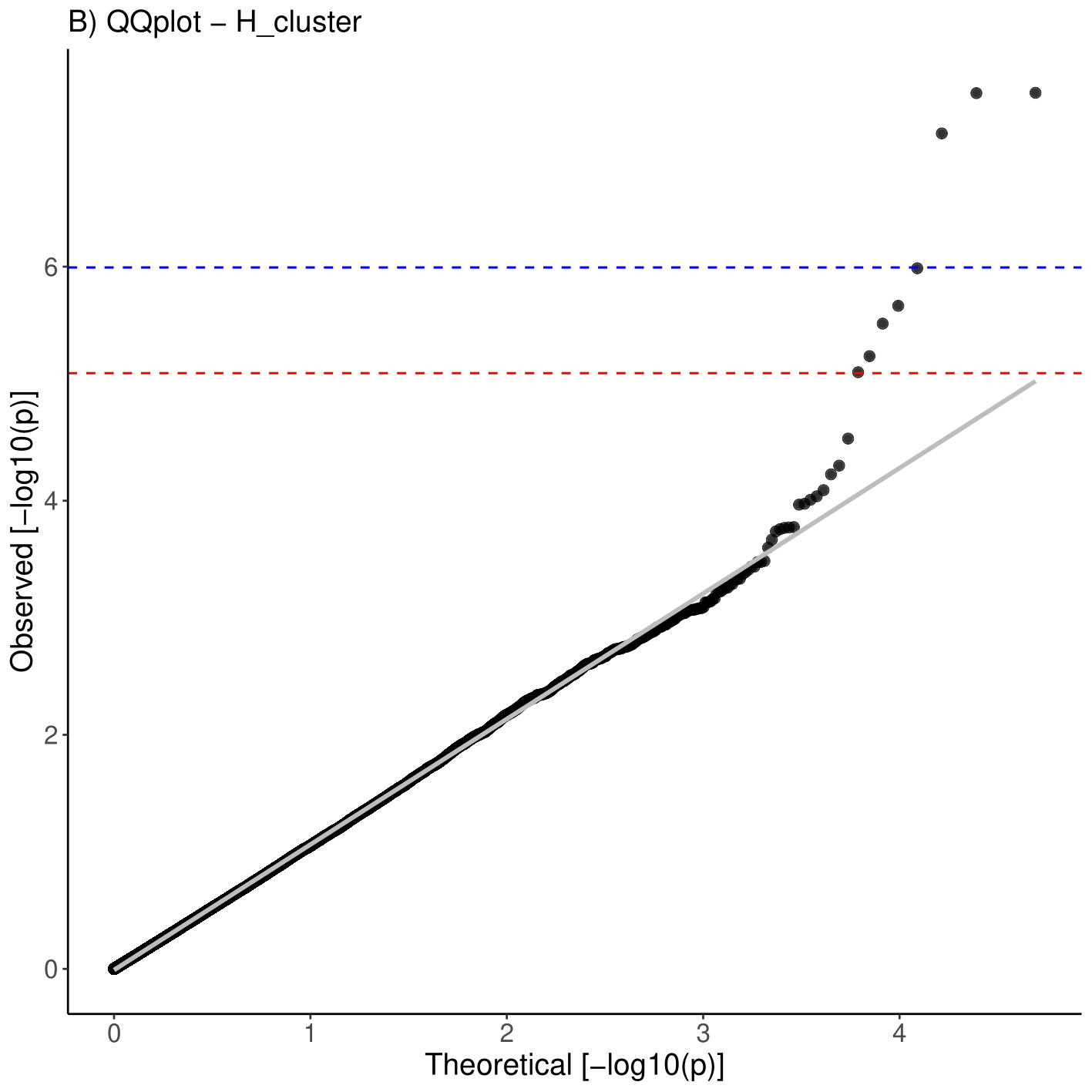


**Supplementary figure S5.** The results of the GWAS analysis for the 13 studied traits were displayed in this figure as Manhattan plots and Q-Q (Quantile-Quantile) plots. The upper row of plots depicted Manhattan plots, which portrayed the physical position of markers plotted against their significance level (LOD score). The blue line was utilized to represent the Bonferroni threshold, while the red line represented the FDR (False Discovery Rate) threshold. Markers with a LOD score higher than the FDR threshold were labelled as red points. The lower row of plots showed the Q-Q plots, which compared the observed LOD scores to the theoretical uniform distribution of LOD scores expected if the null hypothesis of no association were true, represented by the grey line.


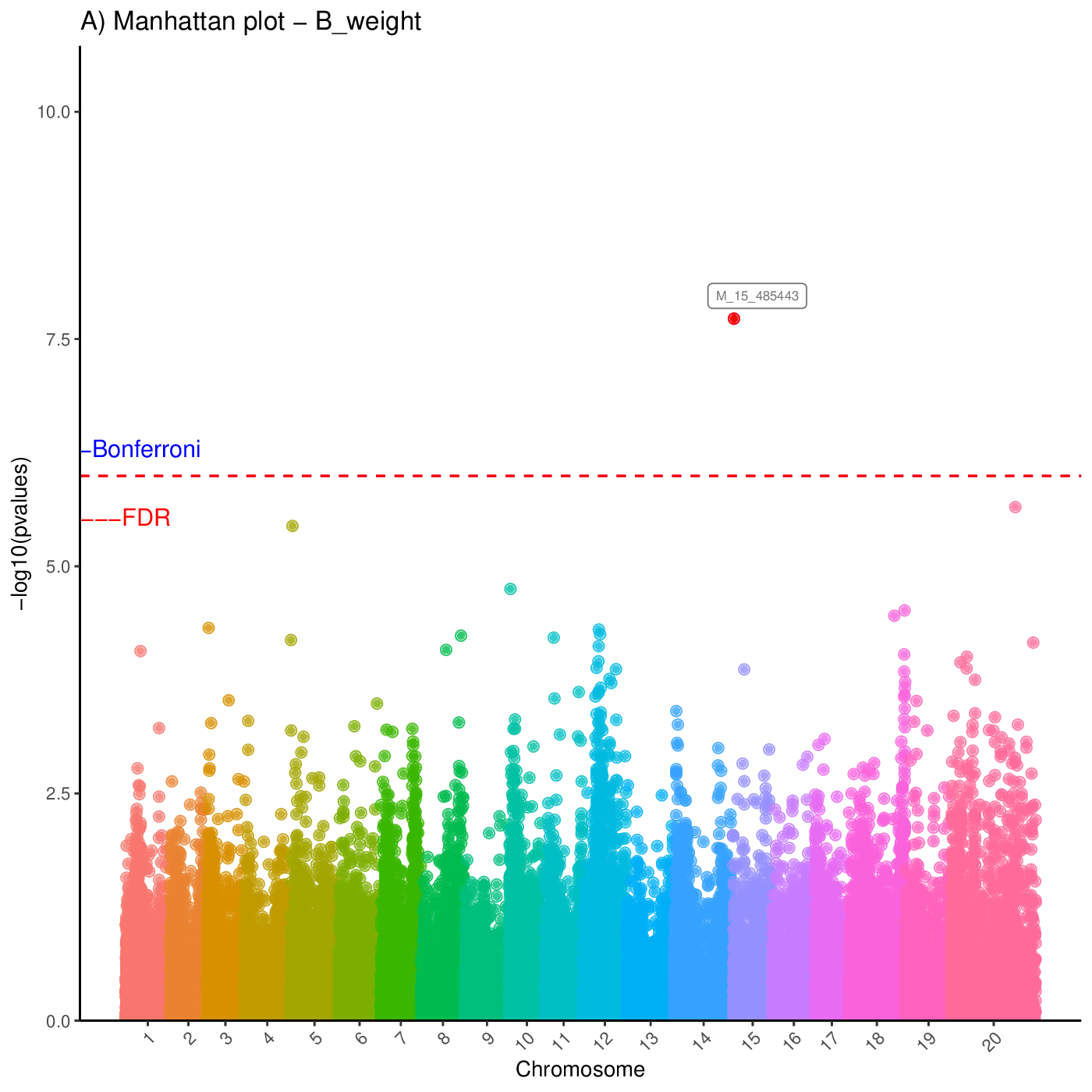

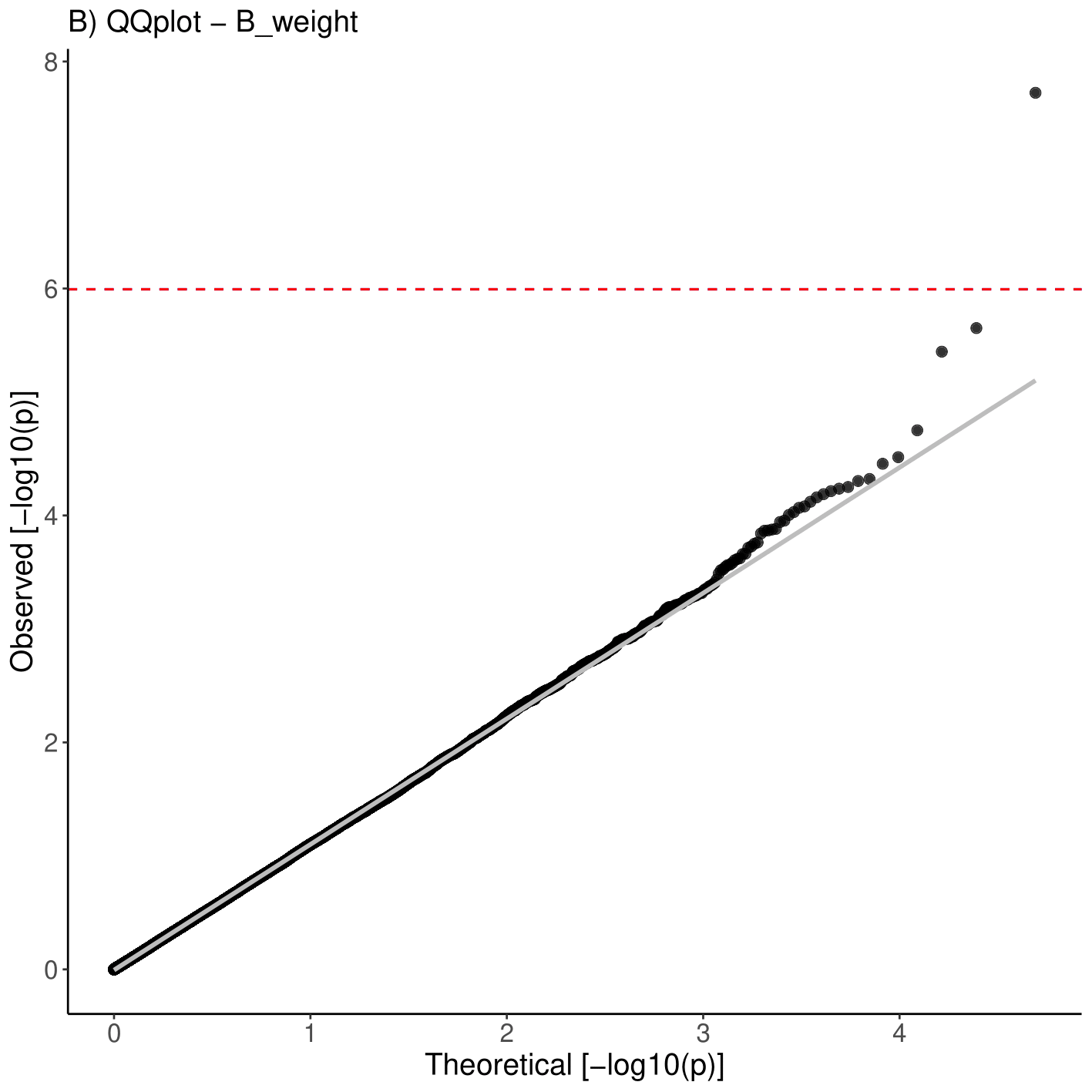

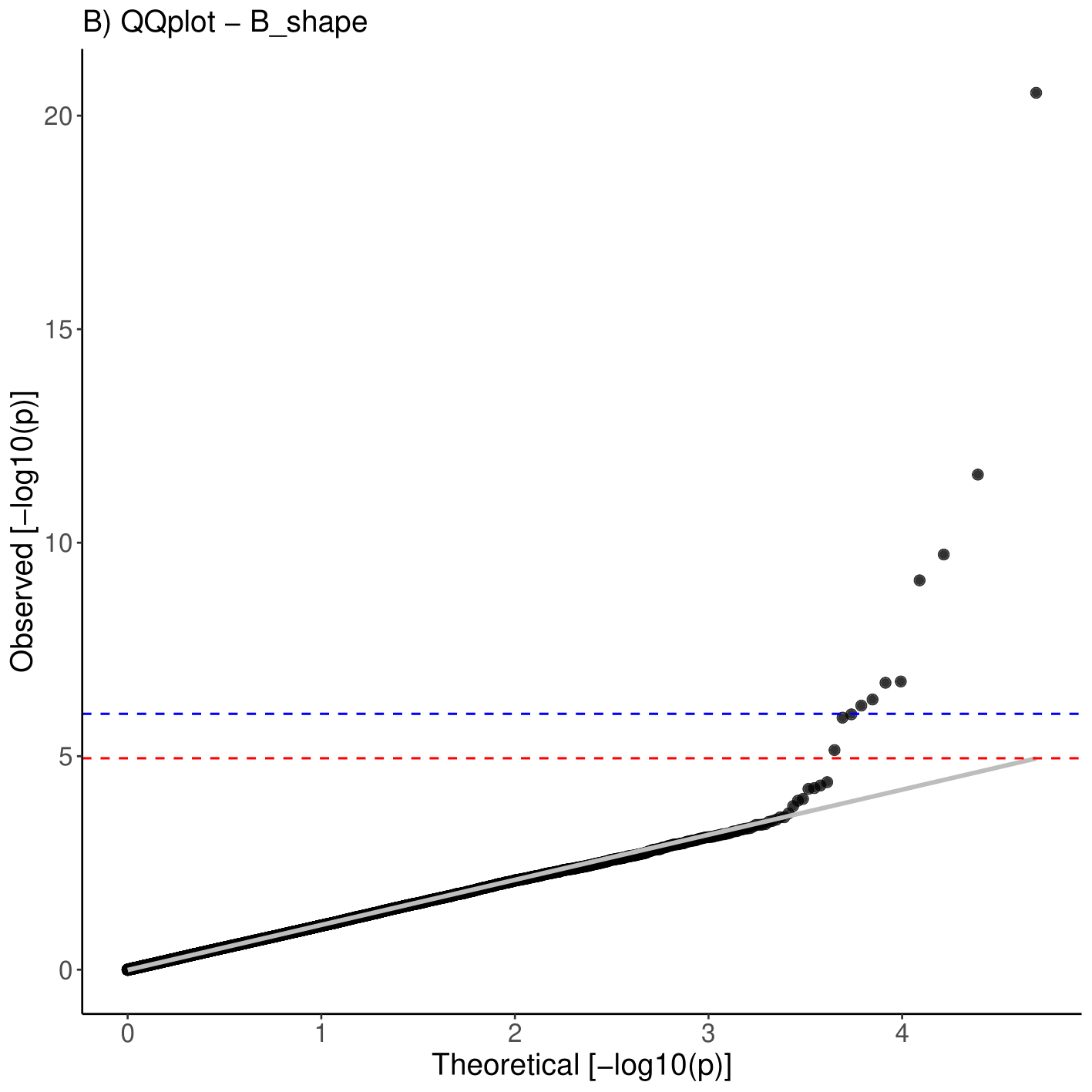

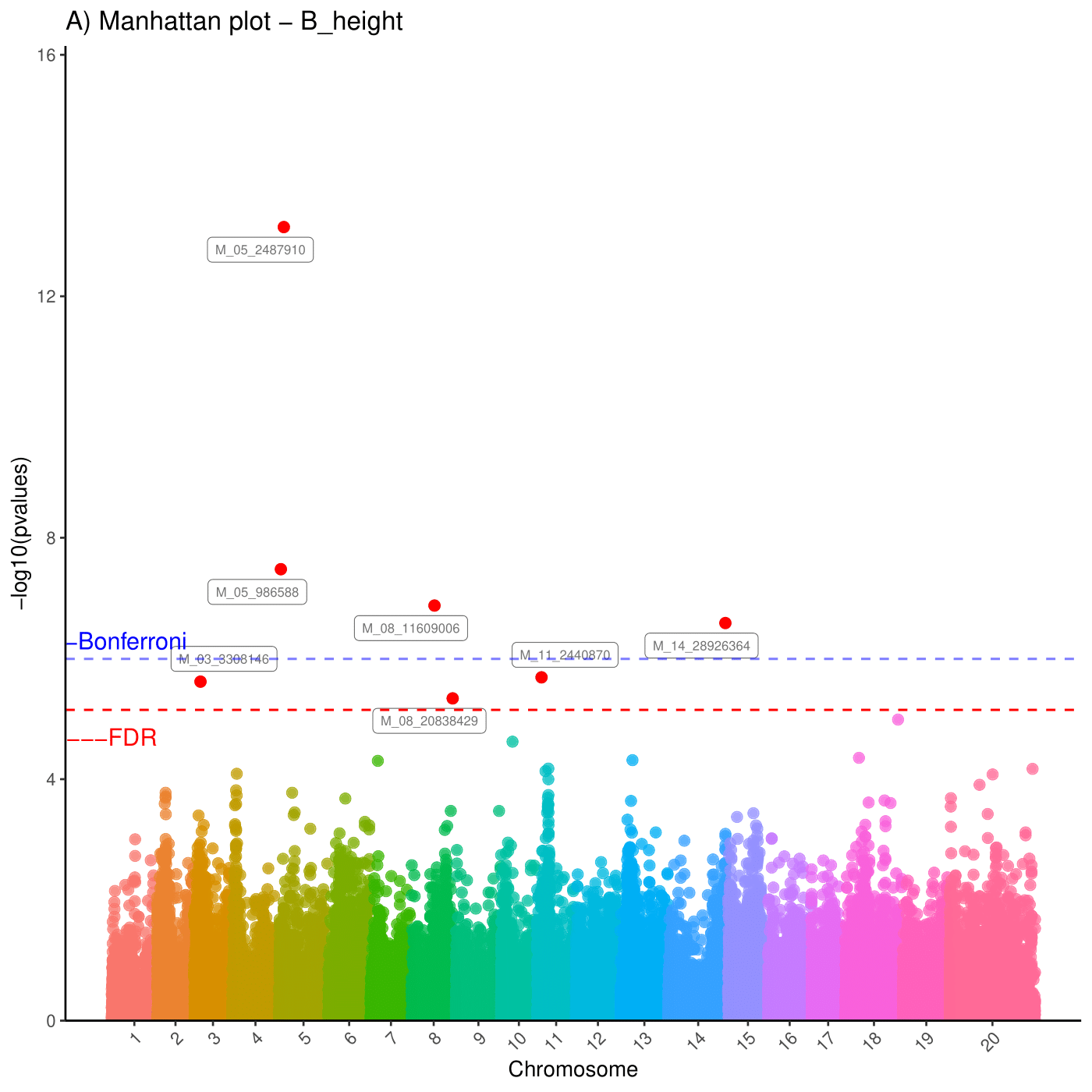

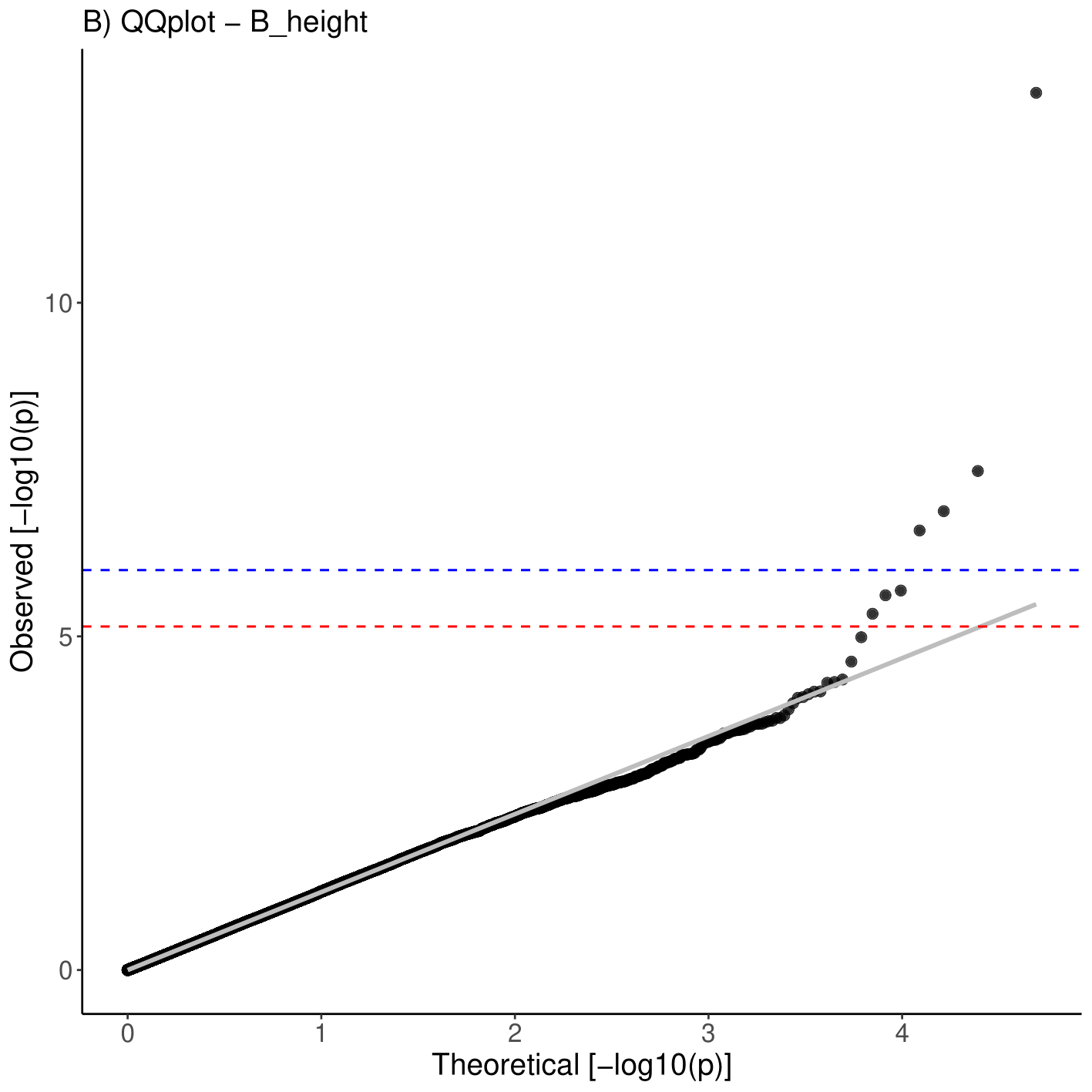

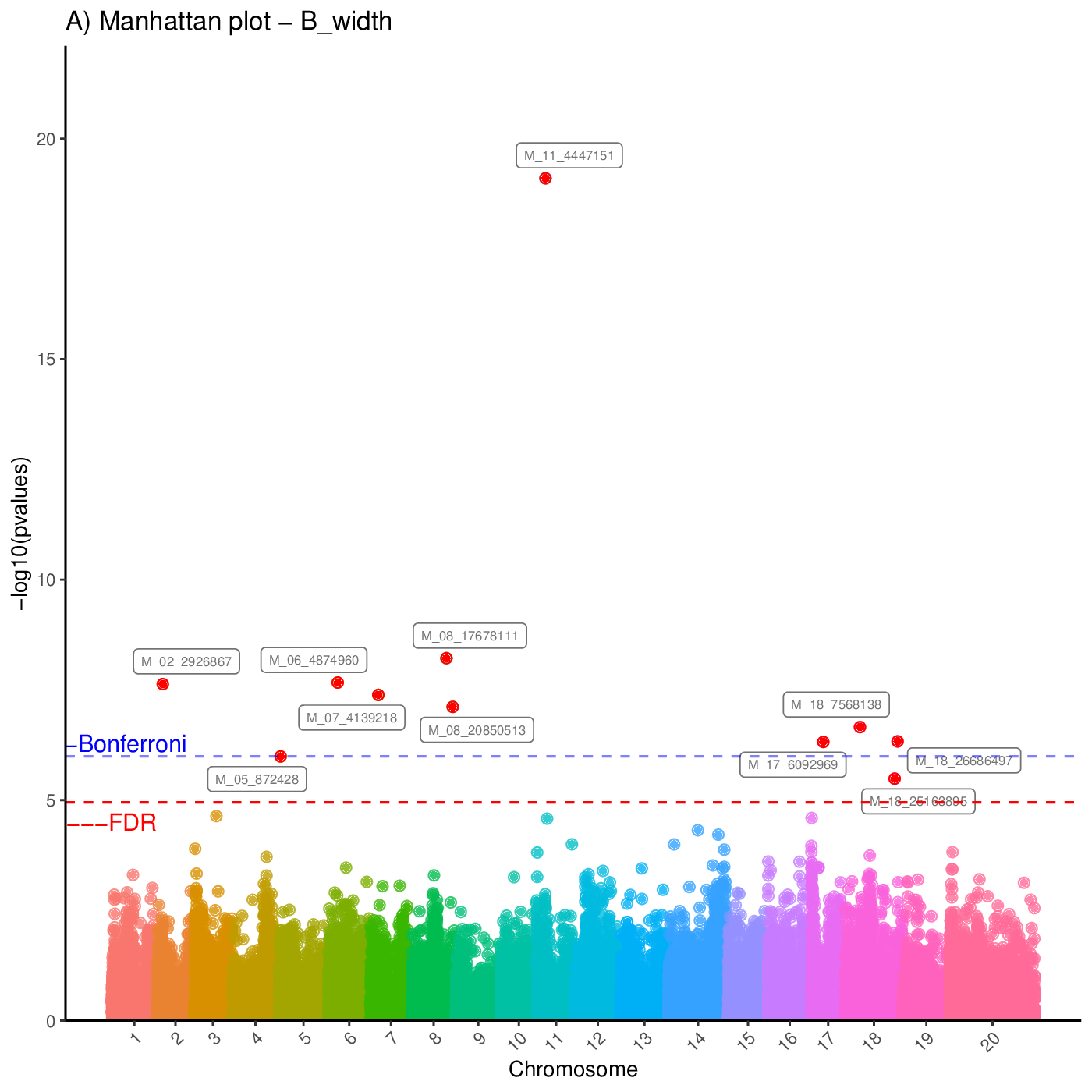

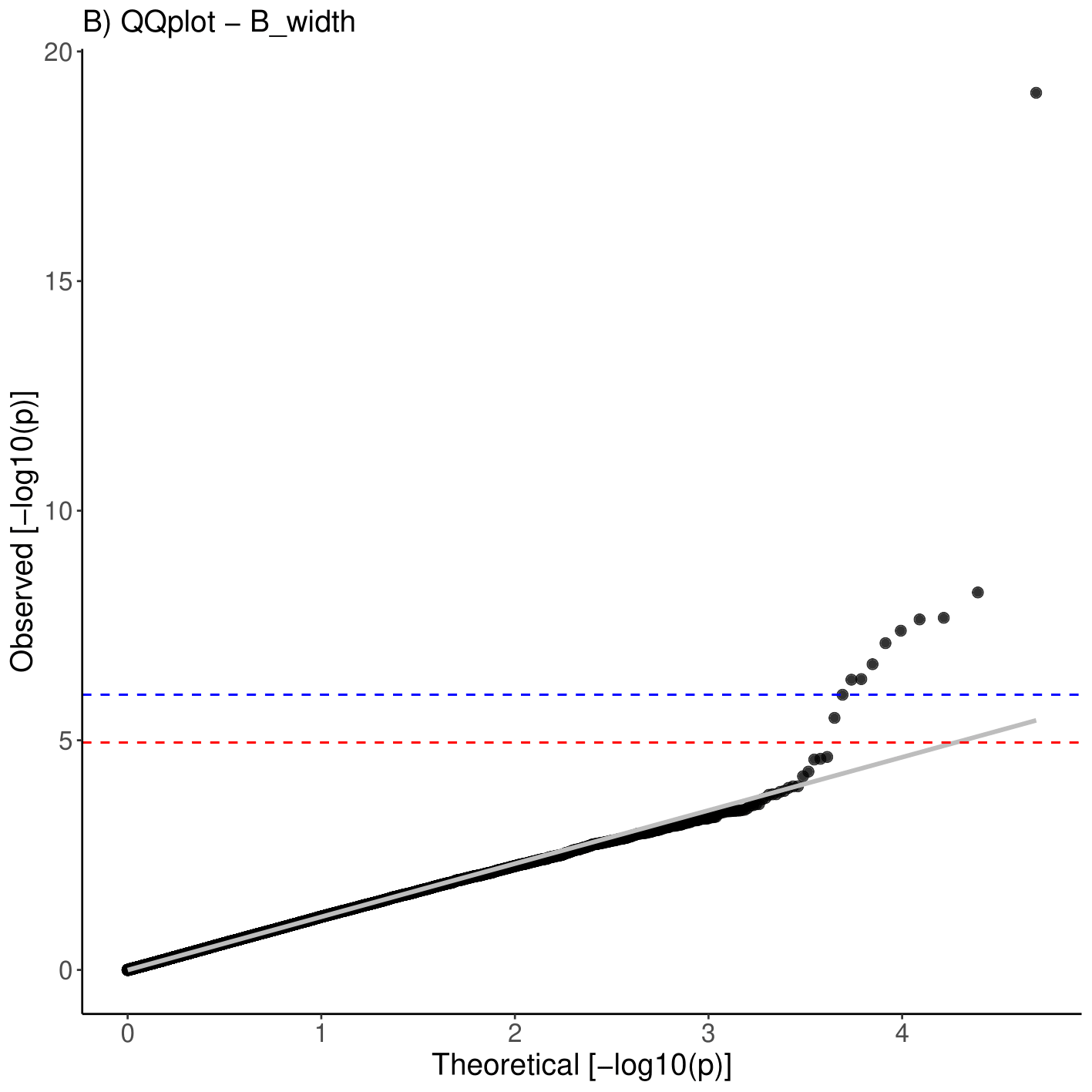

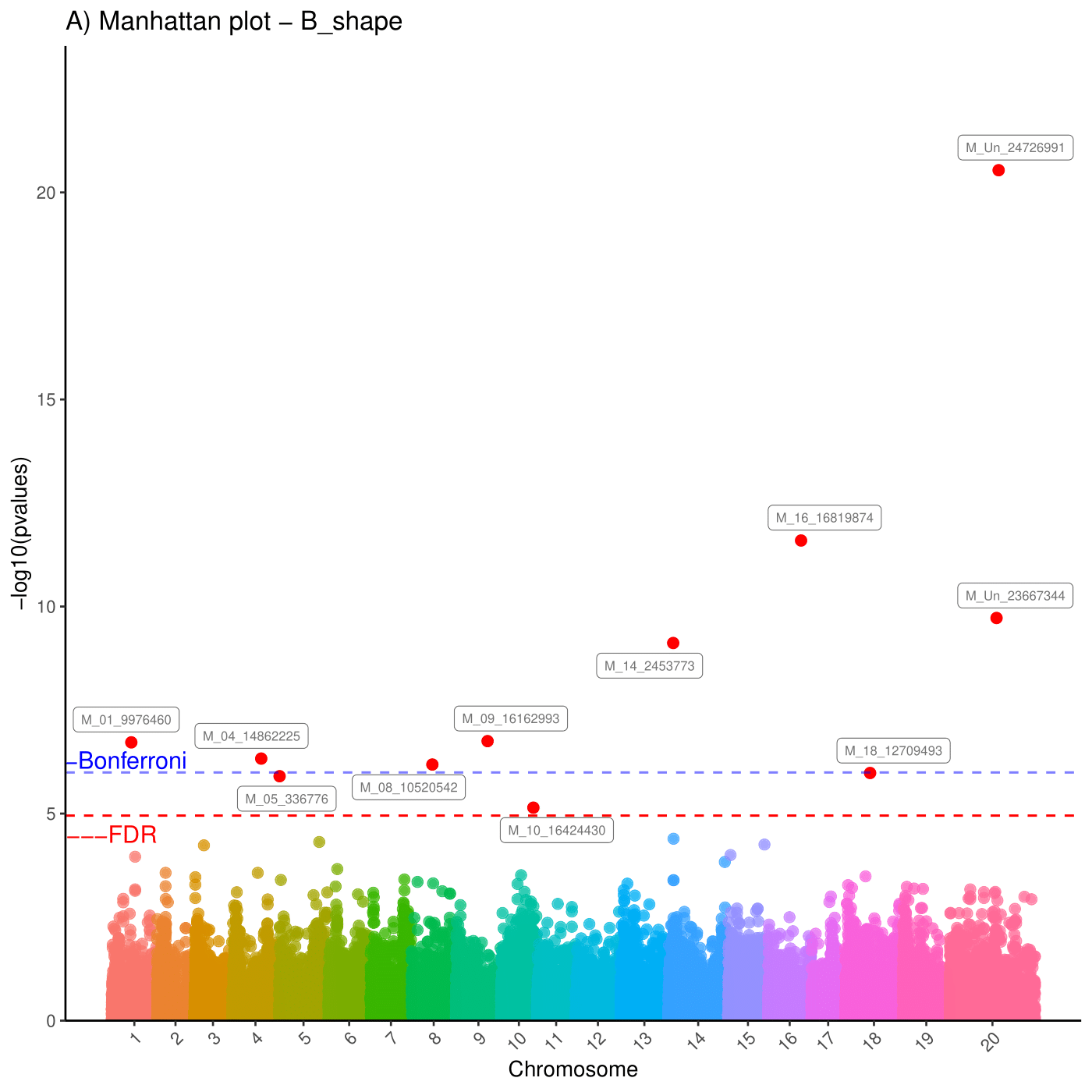


**Supplementary figure S5 (Continue).**


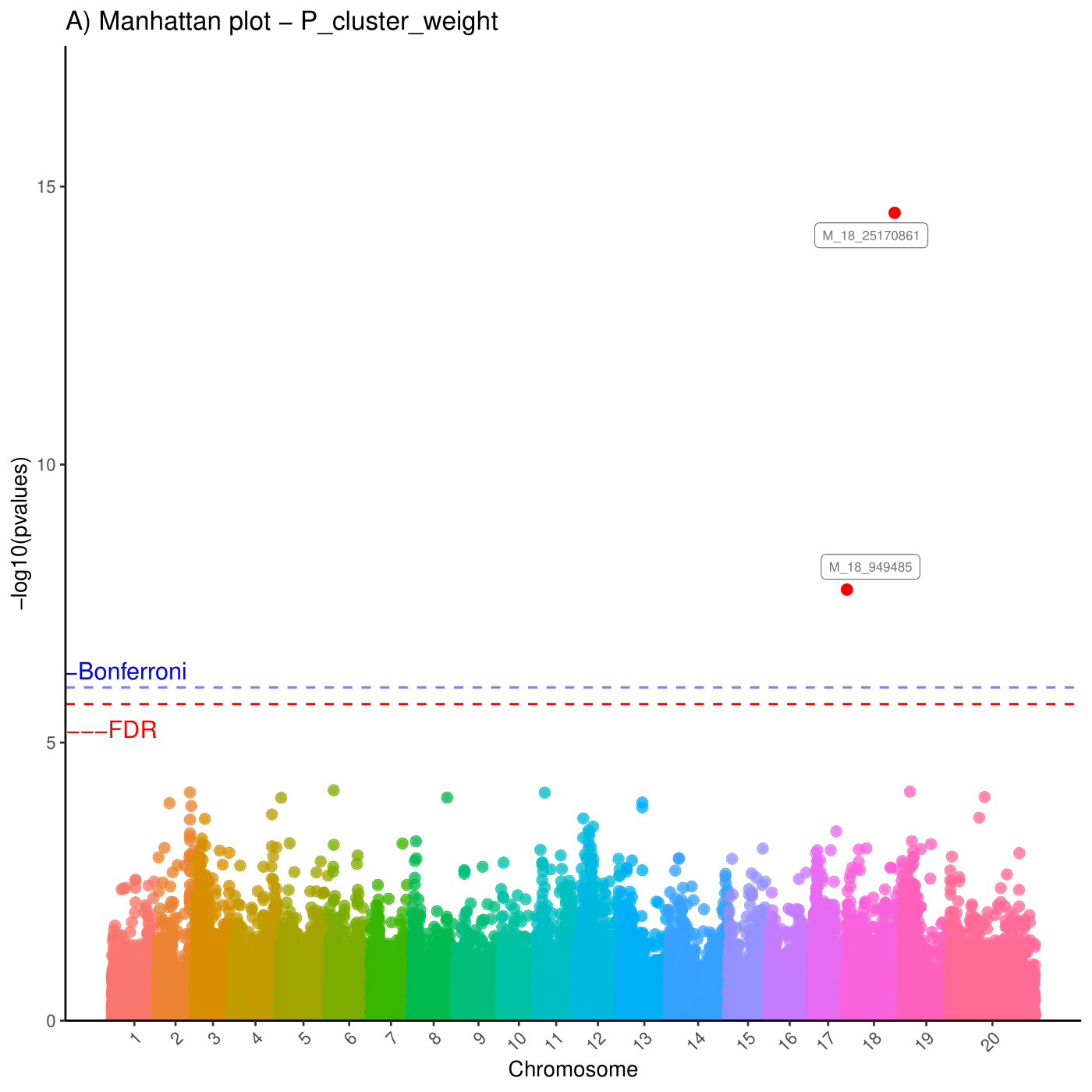

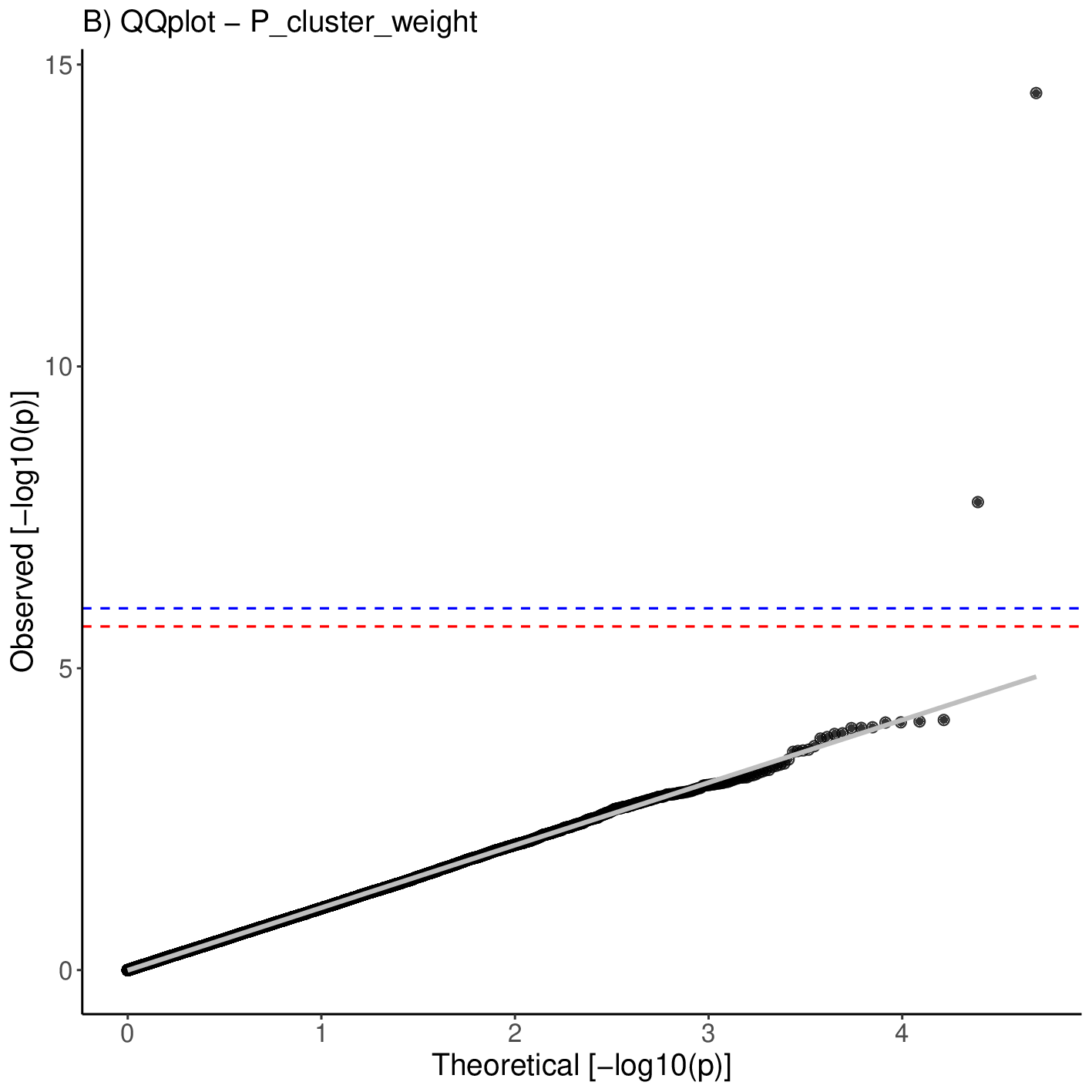

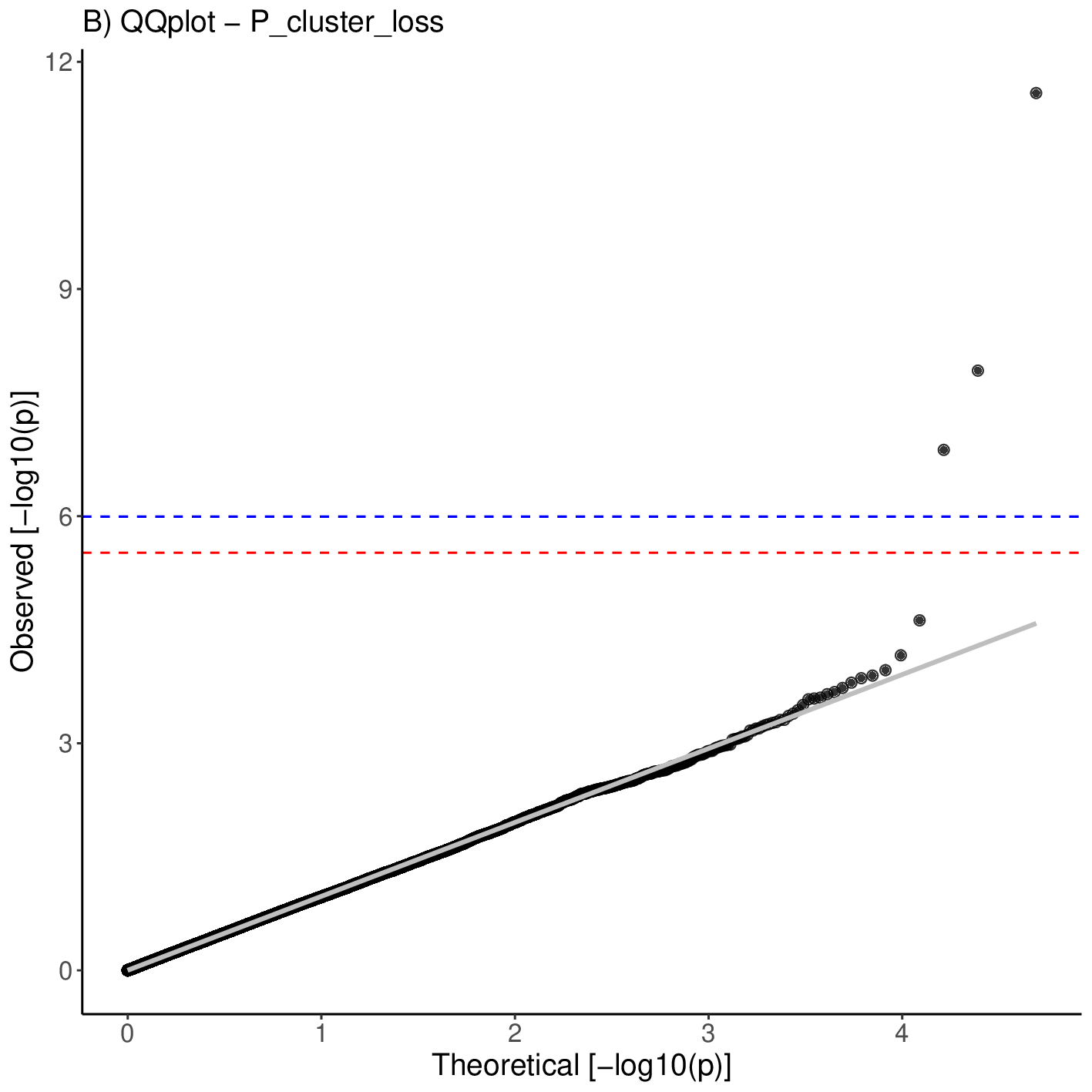

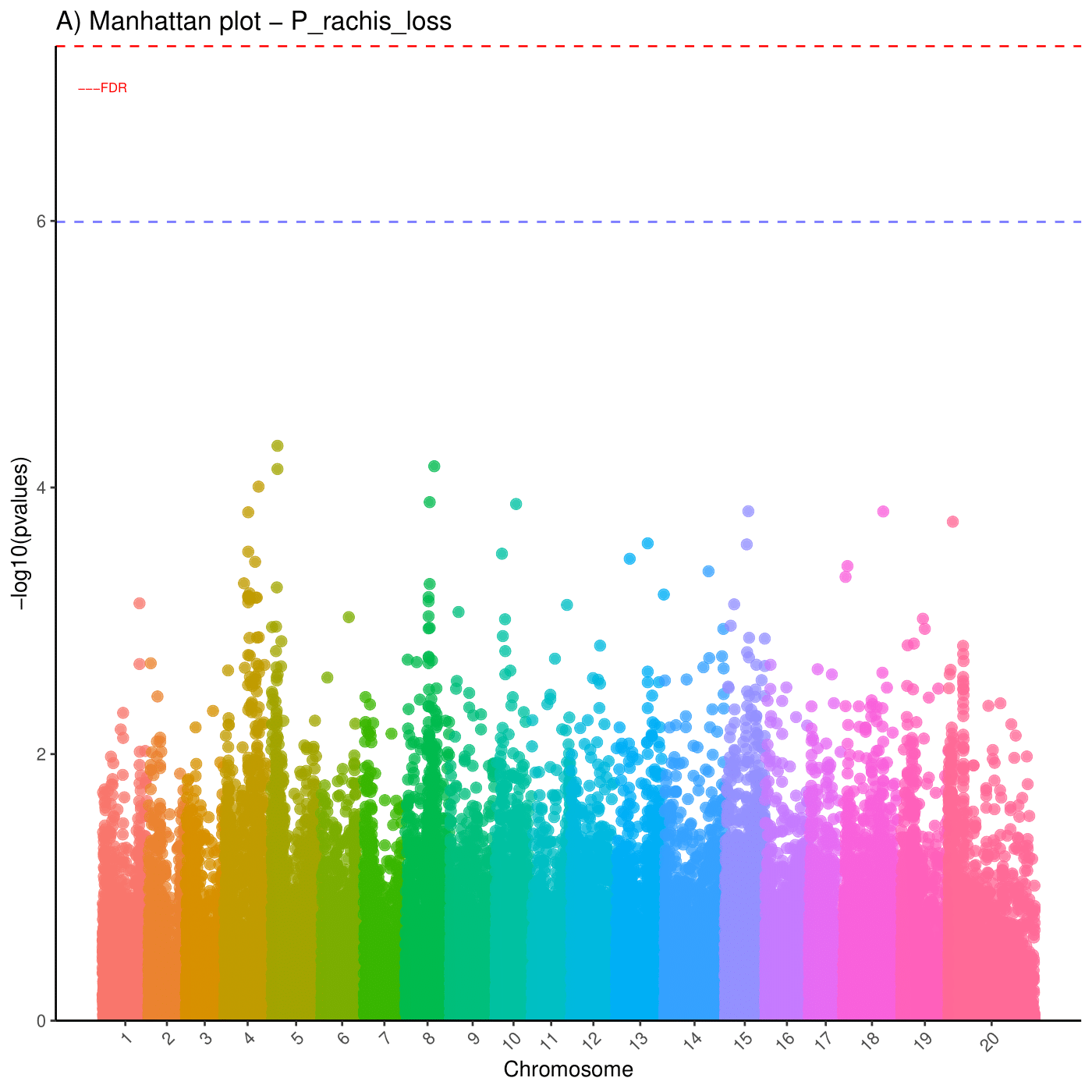

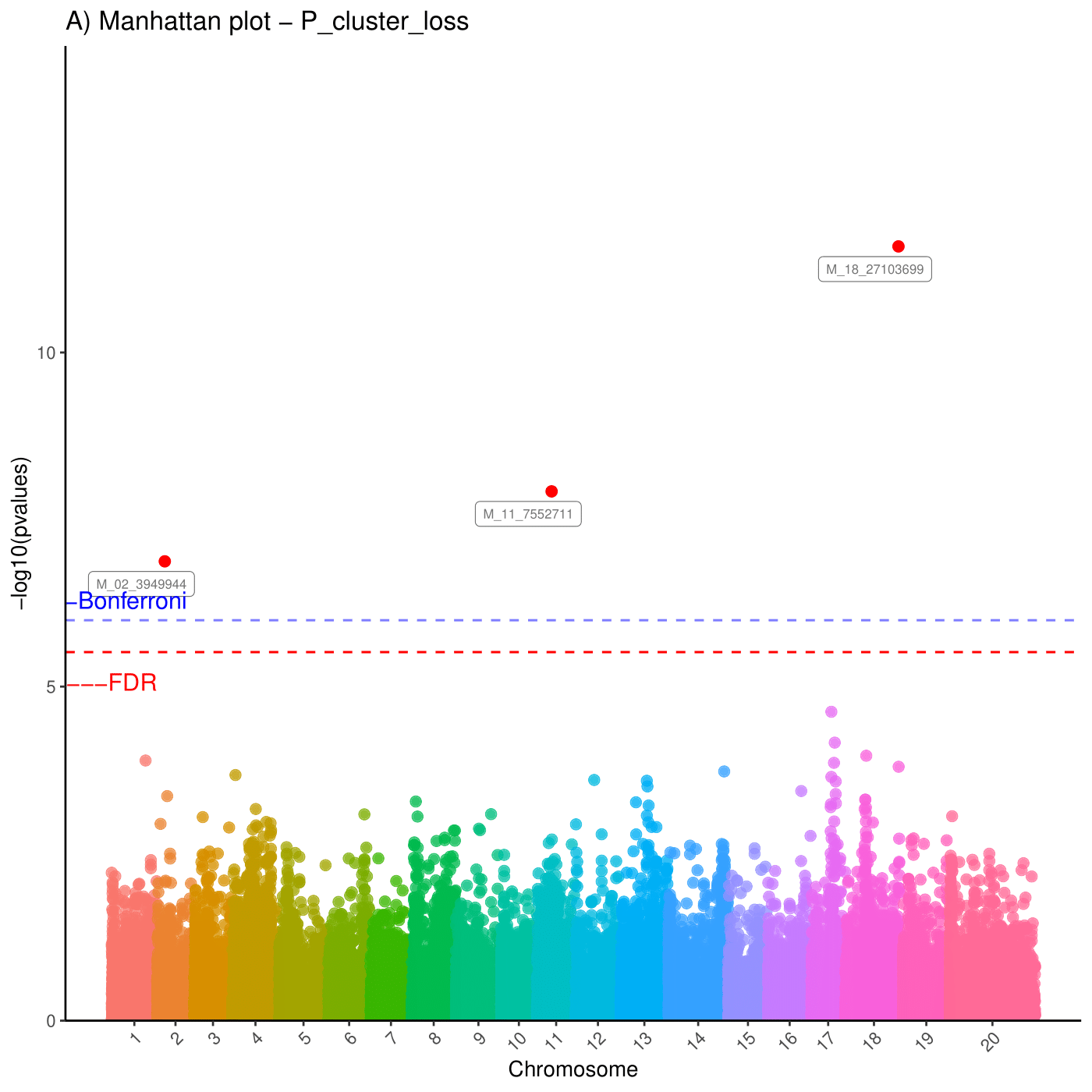

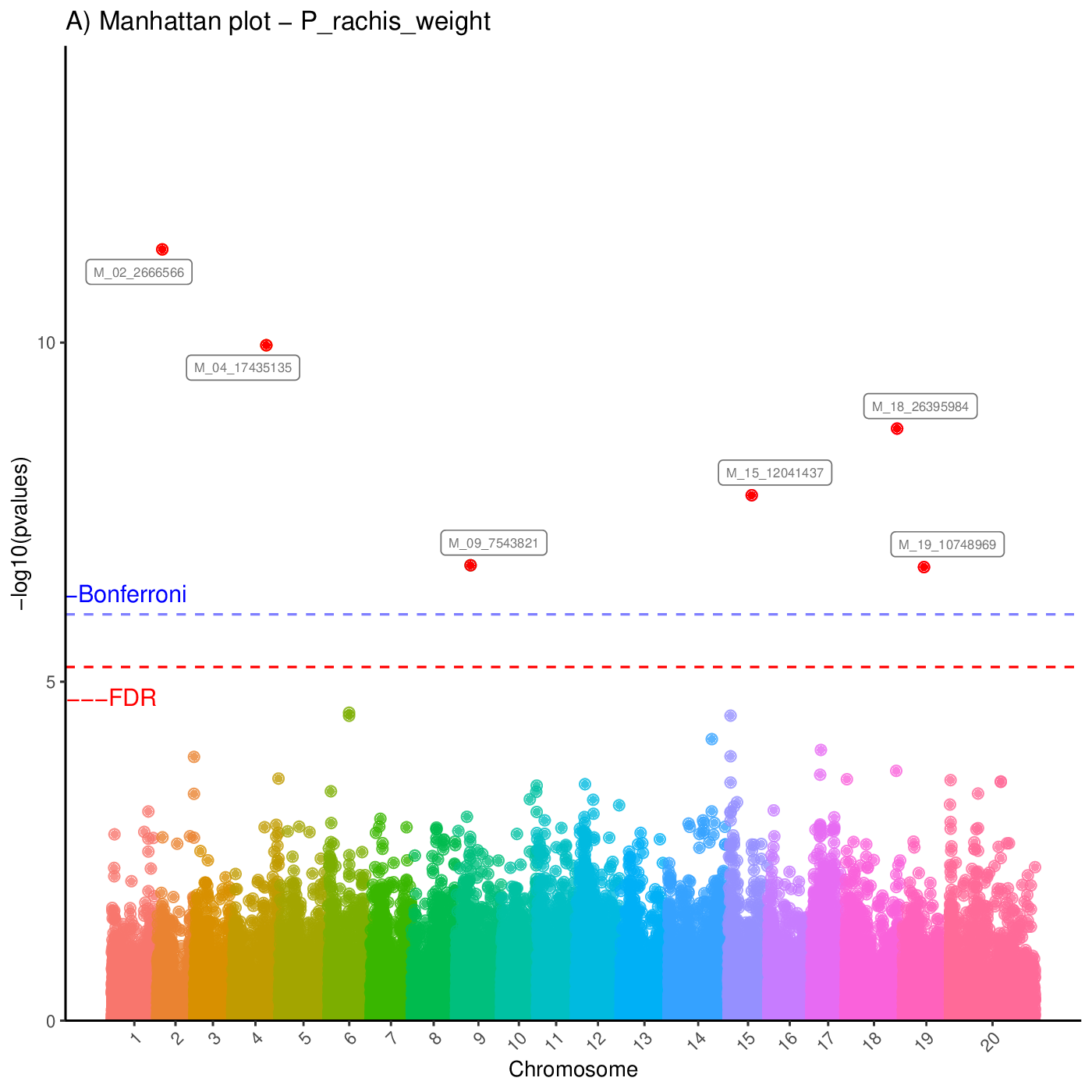

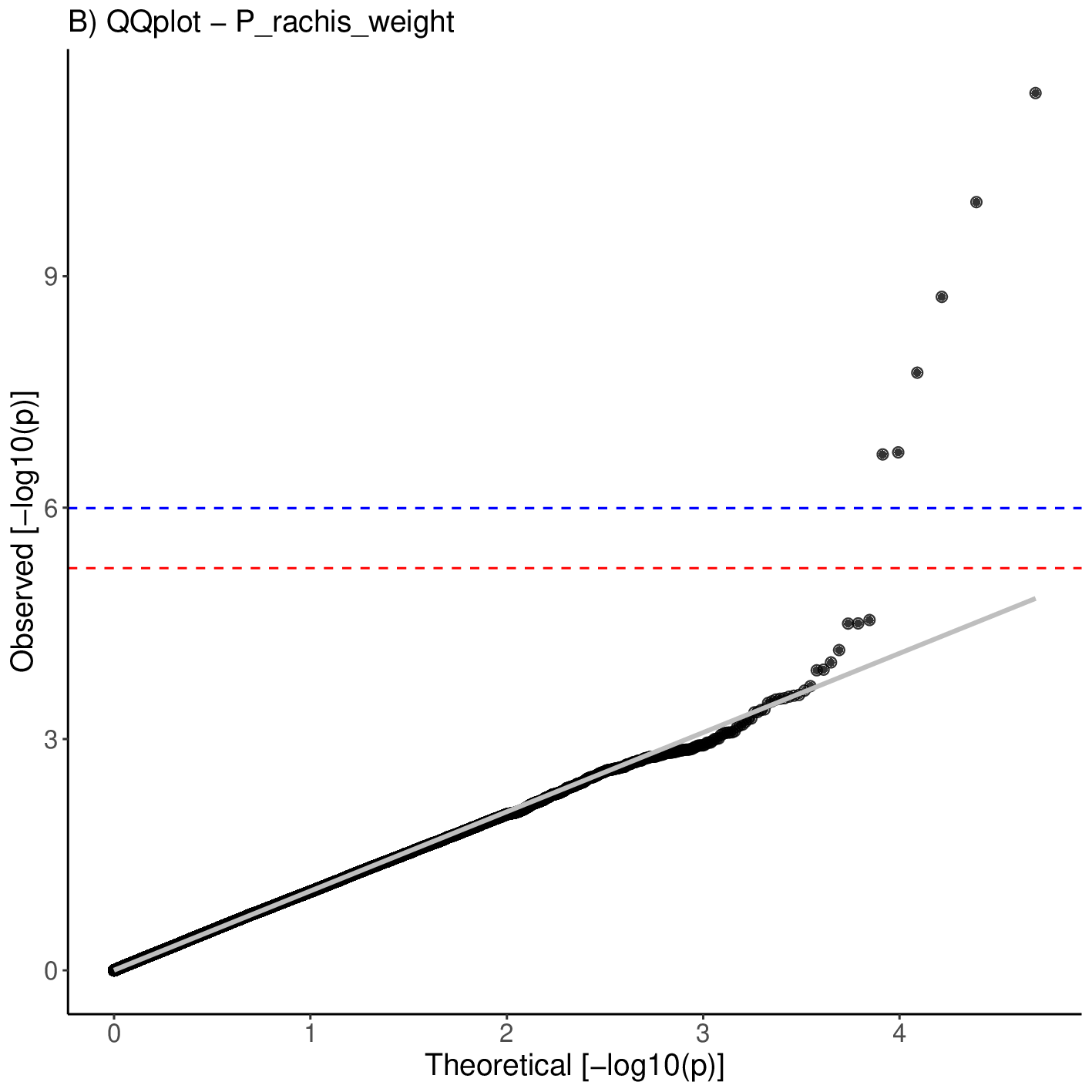

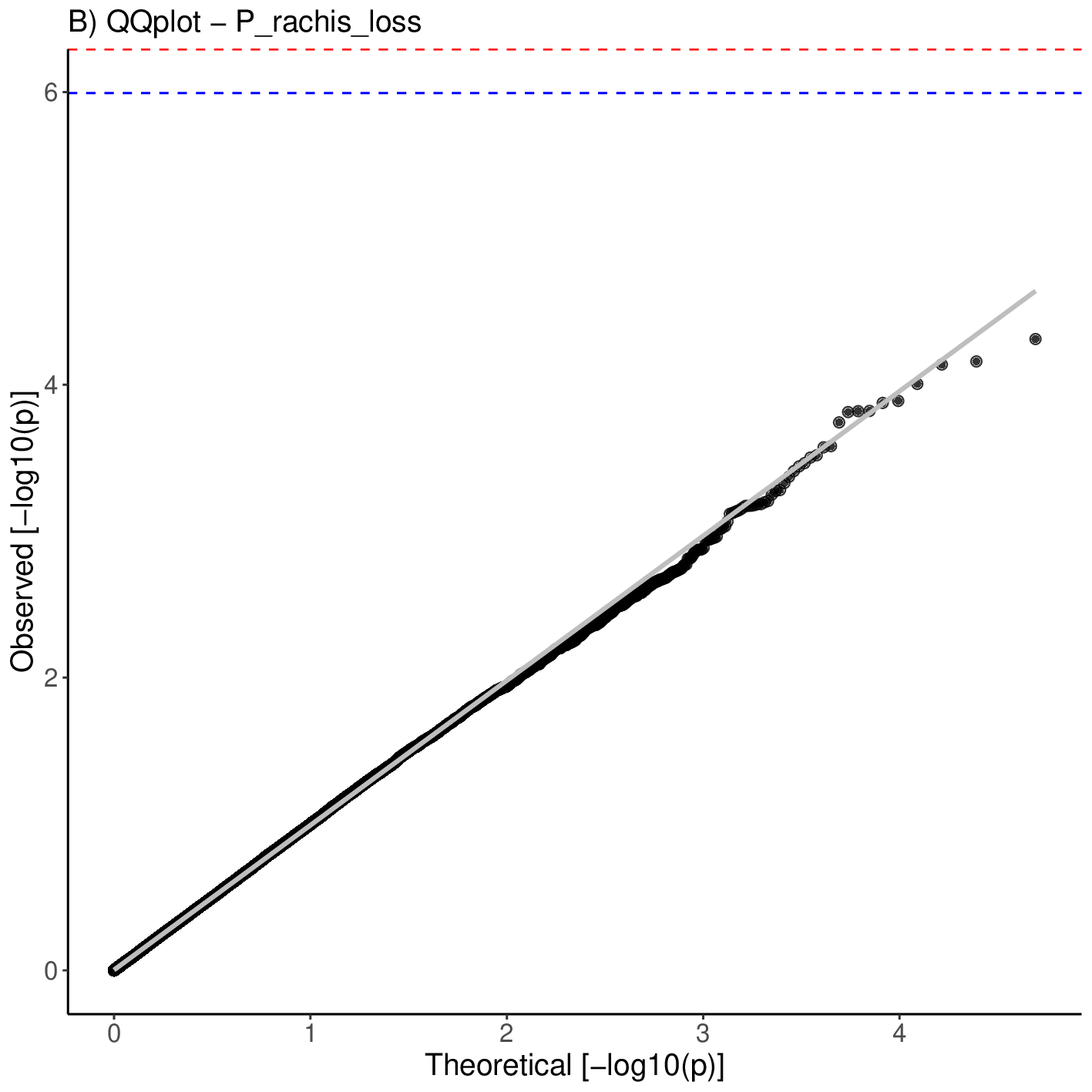


**Supplementary figure S5 (Continue).**

**
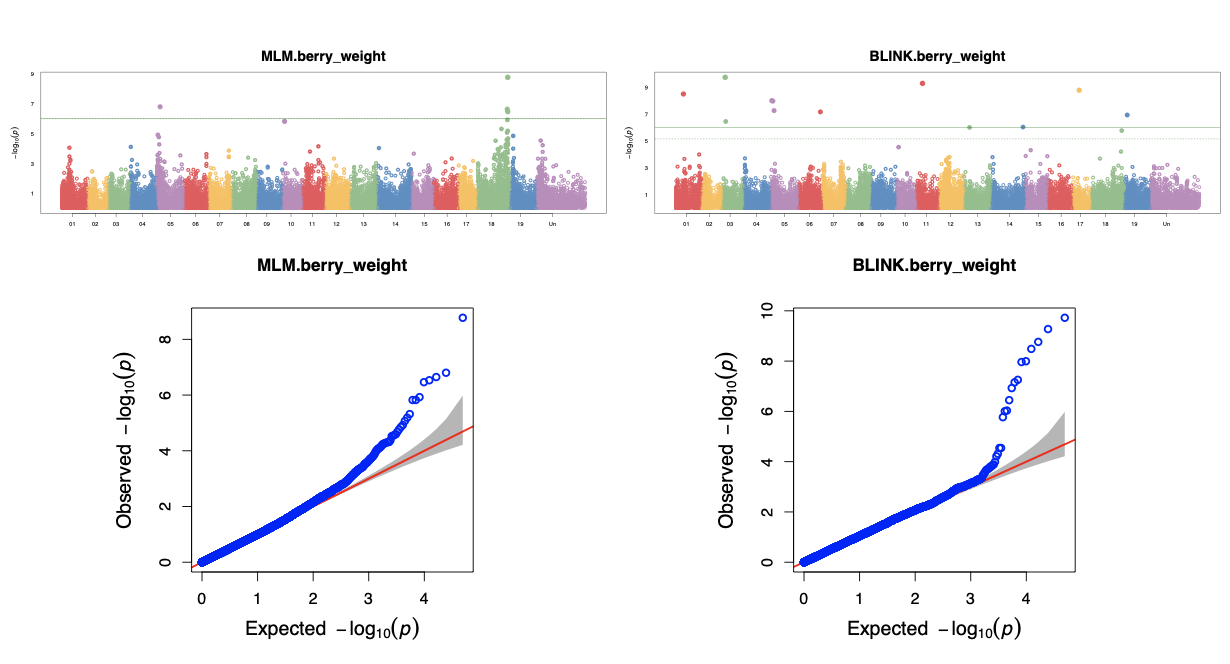
**

**Supplementary figure S6.** Results of the GWAS analysis for berry weight when it is not corrected by seed dry weight. Results of two different GWAS models, MLM and BLINK, are shown in the left and right column, respectively. Manhattan plots are shown in the upper row. Continuous green line represents Bonferroni threshold whereas dashed red line represents FDR threshold. Q-Q plots are shown in the lower row. Red line represents the theoretical LOD scores uniform distribution while blue dots represent the observed LOD scores.

**
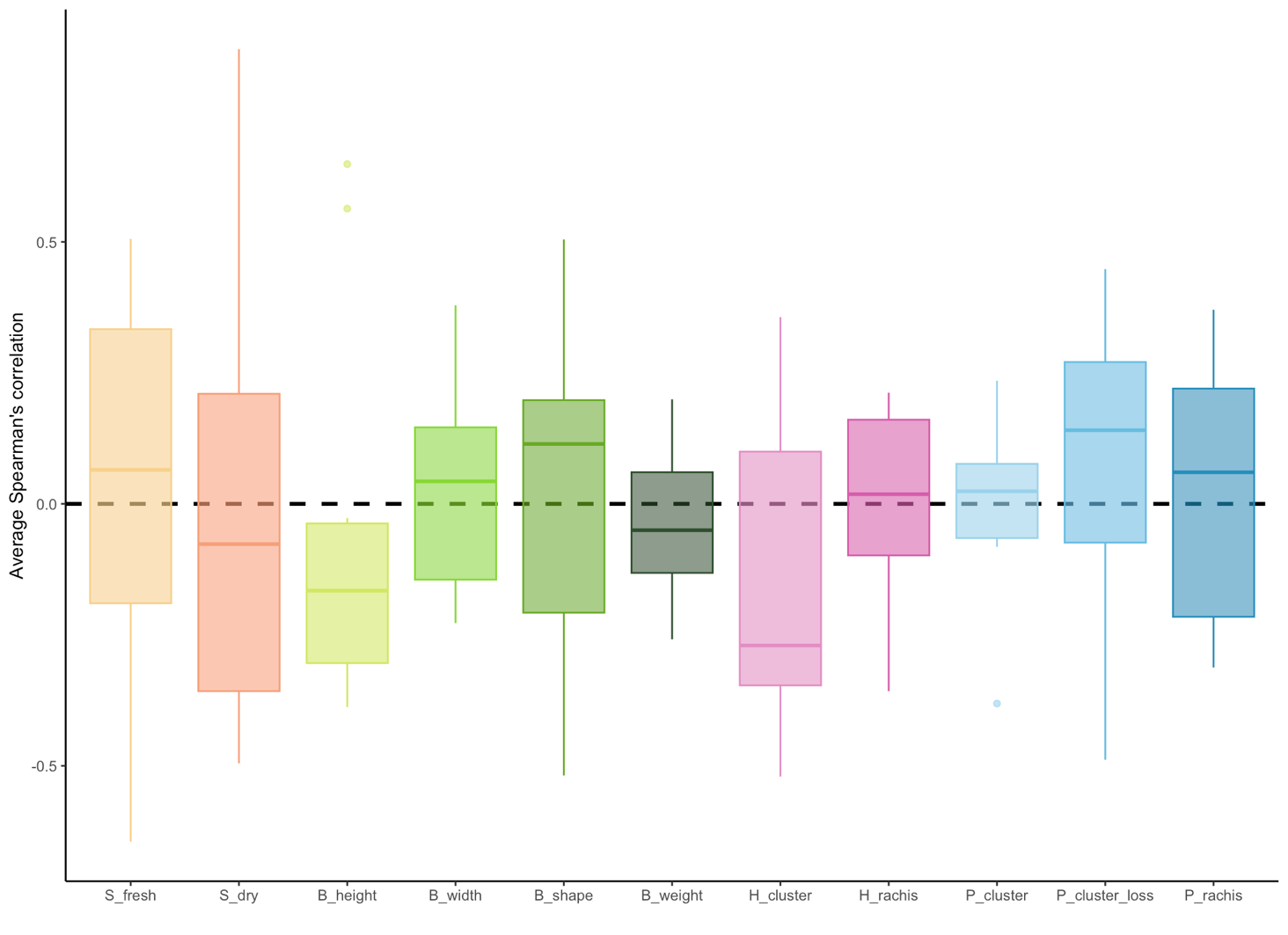
**

**Supplementary figure S7.** Spearman’s correlation distribution of control markers. For each trait, the bootstrapping algorithm described in Section 2.4.1 and Algorithm 1 was applied to 10 non-significant, randomly selected markers with 100 replicates each. X-axis denotes each trait while Y-axis represents the distribution of average Spearman’s correlation (obtained from the 100 replicates) for the 10 randomly selected markers. The black dashed line denotes a null average Spearman’s correlation.


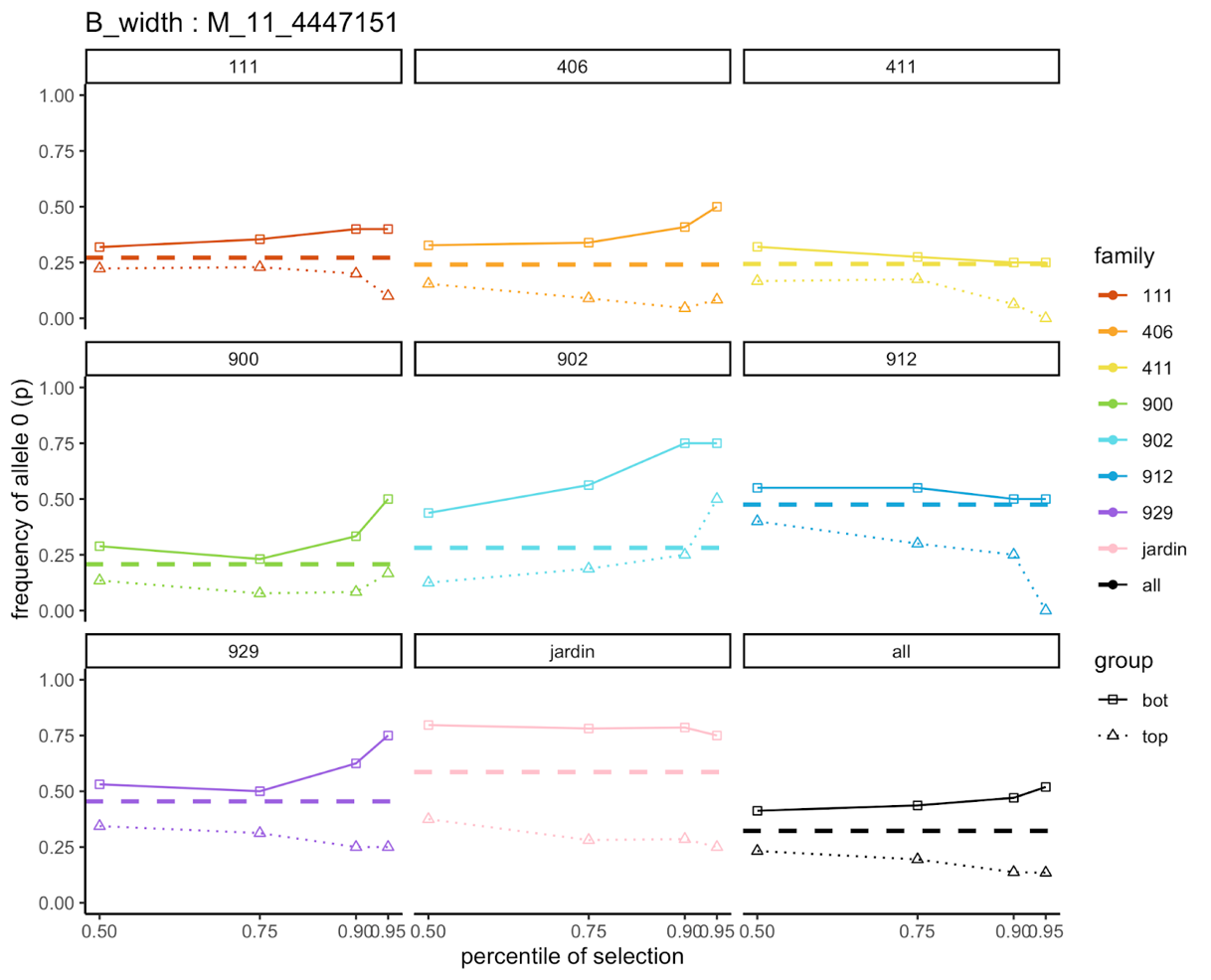


**Supplementary figure S8.** Allelic frequencies among selected populations. Horizontal dashed lines represent the frequency of allele 0 in the whole subpopulations/families. The solid lines correspond to the frequency of allele 0 in the upper population (characterized by higher berry width) while the dotted line depicts the frequency of allele 0 in the lower population (lower berry width). On the x-axis, the percentage of the selected upper/lower group is represented.
